# Supplementary material for: Vocal signatures affected by population identity and environmental sound levels
Source: PLoS One. 2024 Apr 18;19(4):e0299250. doi: 10.1371/journal.pone.0299250 (PMC11025965; doi:10.1371/journal.pone.0299250)
Supplement: Attachment — Submitted filename: Annotated with myComments_PONE-D-23-09444_R1_reviewer.pdf [file pone.0299250.s004.pdf]

PLOS ONE

Vocal signatures affected by population identity and environmental sound levels

--Manuscript Draft--

**Manuscript Number:**	PONE-D-23-09444R1
**Article Type:**	Research Article
**Full Title:**	Vocal signatures affected by population identity and environmental sound levels
**Short Title:**	Vocal signatures affected by population identity and environmental sound levels
**Corresponding Author:**	Amber Fandel<br>University of Maryland Center for Environmental Science<br>Solomons, MD UNITED STATES
**Keywords:**	population identity, bottlenose dolphin, acoustic communication, ambient noise, Tursiops truncatus, vocal signature
**Abstract:**	Passive acoustic monitoring has improved our understanding of vocalizing organisms in remote habitats and during all weather conditions. Many vocally active species are highly mobile, and their populations overlap. However, distinct vocalizations allow the tracking and discrimination of individuals or populations. Using signature whistles, the individually distinct calls of bottlenose dolphins, we calculated a minimum abundance of individuals, characterized and compared signature whistles from five locations, and determined reoccurrences of individuals throughout the Mid-Atlantic Bight and Chesapeake Bay, USA. We identified 1,888 signature whistles in which the duration, number of extrema, start, end, and minimum frequencies of signature whistles varied significantly by site. All characteristics of signature whistles were deemed important for determining from which site the whistle originated. With distinct characteristics and lack of spatial mixing, we suspect that Offshore dolphins are of a different population. Signature whistles were shorter when sound levels were higher. Using only the passively recorded vocalizations of this marine top predator, we obtained information about its population and how it is affected by ambient sound levels, which will increase as offshore wind energy is developed. In this rapidly developing area, these calls offer critical management insights for this protected species.
**Order of Authors:**	Amber Fandel
	Kirsten Silva
	Helen Bailey
**Response to Reviewers:**	Review Comments to the Author<br><br>Please use the space provided to explain your answers to the questions above. You may also include additional comments for the author, including concerns about dual publication, research ethics, or publication ethics. (Please upload your review as an attachment if it exceeds 20,000 characters)<br>Reviewer #1: Comments on Reviewed Manuscript.<br>Title: Vocal signatures affected by population identity and environmental sound levels<br>ID No: PONE-D-23-09444<br>Summary: The manuscript proposes methods to estimate the minimum numbers of the bottlenose dolphins (bd) in the Chesapeake Bay and waters of coastal Maryland and determine differences in the vocalizations of the bd from these sites (if they are site-specific) by comparing them with the vocalizations of bd from other regions. Also, the authors checked if individual bd change their signature in response to ambient sound conditions. Existing models on R are used for the analysis of recorded acoustics data.<br><br>Comments:<br>Kindly find below my comments/suggestions:<br>1. Line 33-33. This looks like an incomplete statement. I would rather suggest you state that passive acoustic monitoring (PAM) is a valuable technique for monitoring vocalizing species. Also, a sentence or 2 to define PAM may be helpful. This open-access review article on marine mammal vocalization may be helpful<br>https://ieeexplore.ieee.org/abstract/document/9110497<br>•Thank you for the suggestion. A sentence on passive acoustic monitoring has been

Powered by Editorial Manager® and ProduXion Manager® from Aries Systems Corporation

added.
“Passive acoustic monitoring uses microphones (referred to as hydrophones in the underwater environment) to detect the sounds of vocalizing organisms.” (line 35-36)
2. There are inconsistencies in the reference style adopted in the manuscript. Some seem APA (line 36 (Janik & Sayigh, 2013)), while others seem to be IEEE line 73 (14), Line 90 (16,17). Authors should stick to one (approved by the journal).
•Thank you for this correction. All citations are now Vancouver.
3. Introduction: a little more details should be given on PAM. The characteristics of dolphins' signatures and how they differ on an individual basis should be provided.
•I have inserted “Passive acoustic monitoring uses microphones (referred to as hydrophones in the underwater environment) to detect the sounds of vocalizing organisms.” (line 35-36) and “These signature whistles comprise more than half of the whistles that wild bottlenose dolphins produce (7,8). They are formed in the first year of the dolphin’s life (9) and remain relatively stable (5,9).” (line 42-44)
4. Figure 1: A clearer map of the study area should be provided. Also, in the explanation of the areas being investigated in this study, the authors should clearly state the two regions on the map. Also, did the author carry out new acoustic recordings for all regions or it uses already available data for some of the sites (as seems to be the explanation on Lines 72-94)? A distinction between sites/regions, as they relate to the manuscript, would make the manuscript clearer to readers.
•The resolution of the map has been increased and I have also underlined the sites from previous studies. I have also included additional details about recordings from previous studies to supplement Table 1. “Previous recordings at Site 1 occurred between June and August in 2017 and July to October in 2018. At Site 2, recordings occurred between July and September of 2016, January to April and June to October in 2017, and June to December in 2018. Site 3 recordings occurred between June and August in 2018.” (line 82-85)

5. What does 14 mean in line 73?
•This was an incorrectly formatted reference. Thank you for the correction.

6. Line 86-87 states: Signature whistles were considered reoccurrences when they occurred on different days or in different locations.
This needs to be explained further.
•I have explained this further so the line reads “When the same signature whistle was identified at a different location or on a different day than the first instance, it was considered a reoccurrence of that individual signature whistle indicating the same dolphin had been detected again.” (line 110-113)
7. Lines 121-130 state that: To understand whether bottlenose dolphins alter their signature whistles in response to……
How were the changes in ambient level determined/measured concerning the selected signature whistles? This needs to be buttressed further (particularly in the discussion of the results).
•To clarify this point, I have moved up the sentence at the end of this paragraph so it now reads “Ambient sound levels were calculated in MATLAB (MathWorks, Natick, Massachusetts, USA) as the relative broadband (up to 24 kHz, given the sampling rate of 48 kHz) sound pressure level (SPL; dB re 1μPa root-mean-square (rms)) during the recording in which the signature whistle occurred (two or five minutes in duration). Sound levels were also calculated for the duration of each acoustic deployment to determine overall ambient sound levels.” (line 159-164) and in the results “When changes in signature whistle characteristics were modeled in relation to the ambient sound levels during which they occurred, only the duration of the signature whistles significantly varied in relation to changing ambient sound levels, decreasing as sound levels increased (Estimate = -0.005, S.E. = 0.003, Wald = 4.08, p = 0.04). All other signature whistle characteristics did not vary between reoccurrences when sound levels varied. (line 263-268)
8. Results section: Lines 134-137 need further clarification, with further details on the results in Table 1.
•We have added context to these lines to read: “A total of 1,172 unique signature whistles were detected at coastal Site 1, 327 at coastal Site 2, and 19 at Site 3 in the Bay (18). A total of 333 unique signature whistles were detected at Site 4 in the Potomac River in the Chesapeake Bay, and 37 at the offshore site, Site 5 (Table 1). An individuals’ dolphin’s presence is indicated by the presence of its signature whistle, so these counts of unique signature whistles represent a minimum abundance of

bottlenose dolphins occurring at each site." (lines 174-179) and "For the subset of signature whistles for which whistle characteristics were measured Table 1), the distribution of these characteristics (start, end, maximum, minimum, and delta frequencies, duration, number of local extrema) were non-parametric (Shapiro-Wilk test; $p < 0.05$)." (lines 186-189)
9. Figures 2-6: Clearer figures (particularly the labels & values) should be provided. The present Figures are not readable!
•Thank you. We have updated the figures.

10. Line 149: sqrt transformation, write in full at 1st mention.
•Thank you. We have spelled it out.
11. Line 152 7 others that follow. What are the units of the frequencies and other parameters?
•Thank you for this question. These numbers are the result of the MANOVA tests, not the values indicating the varied frequencies, therefore it is not appropriate to include units here, but we have referred to them in the Figures.
12. Lines 295-298: from 'These findings…..to the end. The statement looks ambiguous and incomplete. Consider rephrasing with the clear meaning of the intended message. In motivating the need for effective management of the ocean due to increasing activities, more details should be provided by citing relevant related literature.
•We have rearranged this section to read: "Increasing sound levels in the ocean may continue to cause changes in dolphin calls leading to information loss (51) and reducing cohesion amongst dolphin groups, including mother-calf pairs, who use signature whistles to facilitate reunions (8). Sound levels in this region will rise due to development of offshore wind energy, coastal development, bridge construction, and increasing vessel traffic. It is particularly important to understand how individuals from particular populations may be exposed to sound from various human activities and hence how they may be impacted by masking of calls and potential disruption to their communication that could affect their fitness. The use of signature whistles to track individuals can also aid in determining how long individuals take to return to an area and resume normal behaviors after exposure to elevated sound levels. Such information will be vital for management of this region to determine the potential effects of, responses to, and recovery from anthropogenic sound pollution (50)." (lines 339-352)

Reviewer #2: I appreciate the dataset that was curated for this manuscript and know all of the hard work that goes into these sorts of large data analyses. Unfortunately, I do not recommend this manuscript for publication at this time. The main reasons are with the methodology: the methods are not the same for all sites, the methods are not described in enough detail for SIGID, Artwarp thresholds, how many of the same whistle type were allowed at each site, the random forrest classifiers used to classify bottlenose from common dolphins whistles, and the low frequency sampling rate assessing things like maximum whistle frequency and broadband ambient noise levels. I think that with some care there can be some interesting findings from the robust dataset if the authors are able to consider these points.
Line Item Edits

Line 35: This is worded very strangely, consider reworking to just say that marine mammals are very vocal.
•Thank you. We have rephrased as suggested. "In the marine environment, one of the most vocal groups of organisms are marine mammals, which produce a variety of calls." (lines 36-39)
Line 38: Caldwell and Caldwell 1965 founded signature whistles not Sayigh 1990
•We have corrected this, thank you.
Line 80. Why were all of these methods different? 8:2 and 9:1 duty cycle, visual observations compared to the whistle and moan detector?
Please include your own methods on identifying signature whistles from recordings using SIGID and Artwarp. These methods need to stand alone without needing to refer to other papers for operational definitions and thresholds used. For example what similarity threshold did you use to consider a signature whistle a reoccurrence.
A 48 kHz sampling rate gives a 24kHz Nyquist cut off. Dolphin whistles can range far above this range (up to over 40kHz). This needs to be clearly identified as a limitation of the study and may have resulted in distorted whistle feature data.
•These methods varied because the needs and recording conditions at different sites

and time periods varied. The memory/battery capacity of the different recorders that were deployed in the shallower Bay waters compared to the deeper offshore site and the required deployment period between recovery cruises dictated the necessary duty cycle. We have clarified that some sites also had longer deployments, so recording needs varied.
o"Due to longer deployments at Sites 4 and 5, , the Snap was duty-cycled for two minutes on and eight minutes off at Site 4 and one minute on and nine minutes off at Site 5.." (lines 92-94)
We have also clarified that observers were only present at Site 4, so those criteria were only used for that site. At all sites, methods for narrowing down hours with possible signature whistles were used- whether focusing on the days with visual observations or use of the Whistle and Moan Detector.
"At Site 4, a subsample of days were analyzed because of the high rate of dolphin presence at this location. At Site 5 (as with Sites 1 and 2), the PAMGUARD Whistle and Moan Detector (19) was utilized to determine hours with possible dolphin presence and these hours were then manually searched for signature whistles with high signal-to-noise ratios." (lines 94-99)
•We thank the reviewer- we have included the frequency limitation as a caveat in the Discussion. "Additionally, because sounds with frequencies above 24 kHz could not be detected in our recordings, higher frequency signature whistles would have been missed in our analyses [39]." (lines 318-320)
Line 84 you switch to using numbered citations instead of name and date?
•We have remedied this error.
Line 94: I am concerned about the species classification portion of this. You say you used ROCCA for species classification but it performed poorly (no data) and then say you used random forest models to confirm species identity but don't provide any information on those models, their previous success rate or how you confirmed that those were successful here.
When you identified a signature whistle, did you analyze multiple iterations of that whistle or just 1 example? What if one dolphin is hanging around one site for a long time whistling, does their one unqiue signature whistle end up contributing much of the whistle data?
•We understand this concern. We have included text below to elaborate on these results.
"During the summer at the most inshore site (Site 1), bottlenose dolphins are the most likely species to be detected (25). In recordings from 21 hours on 16 days during the summer at Site 1, the ROCCA algorithm classified 61% of whistles as "Ambiguous" (n = 576), 13% as striped dolphin (Stenella coeruleoalba; n = 121), 11% as bottlenose dolphins (n = 103), 8% as common dolphins (Delphinus delphis, n = 74), 4% as Clymene dolphin (Stenella clymene, n = 39), 2 as Atlantic spotted dolphin (Stenella frontalis, n = 19), and 1% as Pantropical spotted dolphin (Stenella attenuate, n.= 7). Clymene dolphins are typically found in waters deeper than 800 feet, and Pantropical spotted dolphins are not located in the Atlantic Ocean." (lines 121-129)
•Additionally, we have added text to elaborate our methods so this paper stands alone. "Signature whistles were obtained using the SIGID criteria (1)-the same whistle repeated in a pattern of two or more whistles (7) within 1–10 s of one another and with a minimum length of 0.2 s (20,21). Whistle contours (shape of the whistle) were obtained using Beluga software (https://synergy.st-andrews.ac.uk/soundanalysis) within MATLAB (Math-Works, Natick, Massachusetts, USA). Whistles with low signal-to-noise ratio or abundant non-linear features (22) that obscured the shape of the whistle could not be included in the analysis. Beluga contours were analyzed within the adaptive resonance theory neural network (ART-warp; vigilance threshold of 96%; (23)) to identify when a whistle reoccurred. Identical signature whistles were not considered re-occurrences if they occurred on the same day at the same site. A human analyst verified all whistle re-occurrences." (lines 99-108)
Line 125- What is a 'clear' signature whistle? Sportelli et al 2022 found that over 50% of signature whistles have non-linear features… Does this mean you did not include any whistles that had NLP?
•Whistles with non-linear features were included as long as analysts could determine the shape of the whistle. If too many non-linear features obscured the shape of the whistle, Beluga could not be used to obtain a contour, and thus the analysis could not be completed in ARTwarp. We have added text to indicate this. "Whistles with low signal-to-noise ratio or abundant non-linear features [22] that obscured the shape of the whistle could not be included in the analysis." (lines 103-104)

Powered by Editorial Manager® and ProduXion Manager® from Aries Systems Corporation

	Line 129- I am having a hard time with calling the ambient sound levels 'broad band' up to 24kHz when dolphin hearing goes into the 100s of kHz. Arguably there could have been any number of anthropogenic sound sources above the recording range.<br>•In this case, the term broadband was used to indicate that the sound level was calculated over the frequency range of the recorder, not to the hearing range of the species. We have rephrased this to read "as the broadband (up to 24 kHz, given the sampling rate of 48 kHz) sound pressure level" (line 159-161)<br>Discussion- what was the point of identifying signature whistles if the question was based on whistle features changing based on population, location and or ambient noise? A discussion of why not include all whistles would be helpful.<br>• Our research questions were: whether signature whistles varied by site indicating differential habitat use by individuals and potentially delineating populations; and whether animals adjusted their identity signature whistles in response to varied sound levels. Since this focuses on these unique, individually identifiable signature whistles, we did not include other types of whistle in our analysis.<br>If the signature whistle features were significantly different than a random selection of whistles at the same site, wouldn't this be an important result to suggest that the differences between sites may not be due to sites or ambient noise but just based on the fact that the different dolphins have different signature whistles?<br>•This is an excellent observation. However, because we did not compare a random sample of whistles from each site to signature whistles from those sites, we cannot make the conclusion that signature whistles vary from or are similar to other whistles from those locations or in those sound levels. We specifically compared signature whistles between sites and then examined whether there were any differences in repeated emissions of these signature whistles recorded during reoccurrences at other sites and under different ambient sound conditions.<br>Line 276: Your recording devices did not go up to the highest recorded whistle frequencies. See Jones et al., 2020 for a review or Kaplan and Reiss 2017 or Hiley et al. 2017 for evidence of whistles into the 40s of kHz.<br>•Thank you for these citations. We have added a citation to caveat our results. "Additionally, because sound with frequencies above 24 kHz could not be detected in our recordings, higher frequency signature whistles would have been missed in our analyses [35]." (lines 318-320)
**Additional Information:**	
**Question**	**Response**
**Financial Disclosure**<br><br>Enter a financial disclosure statement that describes the sources of funding for the work included in this submission. Review the submission guidelines for detailed requirements. View published research articles from *PLOS ONE* for specific examples.<br><br>This statement is required for submission and **will appear in the published article** if the submission is accepted. Please make sure it is accurate.	The Maryland Department of Natural Resources secured funding for this project from the Maryland Energy Administration's Offshore Wind Development Fund (Contract number 14-18-2420 MEA: HB, ADF, KS: energy.maryland.gov/Pages/Info/renewable/offshorewind.aspx). The views and conclusions contained in this document are those of the authors and should not be interpreted as representing the opinions or policies of the Maryland Department of Natural Resources, or the Maryland Energy Administration. Mention of trade names or commercial products does not constitute their endorsement by the state. The funders had no role in study design, data collection and analysis, decision to publish, or preparation of the manuscript.

**Unfunded studies**<br>Enter: *The author(s) received no specific funding for this work.*<br><br>**Funded studies**<br>Enter a statement with the following details:<br>• Initials of the authors who received each award<br>• Grant numbers awarded to each author<br>• The full name of each funder<br>• URL of each funder website<br>• Did the sponsors or funders play any role in the study design, data collection and analysis, decision to publish, or preparation of the manuscript?<br>• **NO** - Include this sentence at the end of your statement: *The funders had no role in study design, data collection and analysis, decision to publish, or preparation of the manuscript.*<br>• **YES** - Specify the role(s) played.<br><br>* typeset	
**Competing Interests**<br><br>Use the instructions below to enter a competing interest statement for this submission. On behalf of all authors, disclose any competing interests that could be perceived to bias this work—acknowledging all financial support and any other relevant financial or non-financial competing interests.<br><br>This statement is required for submission and **will appear in the published article** if the submission is accepted. Please make sure it is accurate and that any funding sources listed in your Funding Information later in the submission form are also declared in your Financial Disclosure statement.<br><br>View published research articles from *PLOS ONE* for specific examples.	The authors have declared that no competing interests exist.

**NO authors have competing interests**<br><br>Enter: *The authors have declared that no competing interests exist.*<br><br>**Authors with competing interests**<br><br>Enter competing interest details beginning with this statement:<br><br>*I have read the journal's policy and the authors of this manuscript have the following competing interests: [insert competing interests here]*<br><br>* typeset	
**Ethics Statement**<br><br>Enter an ethics statement for this submission. This statement is required if the study involved:<br><br>• Human participants<br>• Human specimens or tissue<br>• Vertebrate animals or cephalopods<br>• Vertebrate embryos or tissues<br>• Field research<br><br>Write "N/A" if the submission does not require an ethics statement.<br><br>General guidance is provided below. Consult the submission guidelines for detailed instructions. **Make sure that all information entered here is included in the Methods section of the manuscript.**	No ethics statements are needed for this study.

**Format for specific study types**<br><br>**Human Subject Research (involving human participants and/or tissue)**<br>• Give the name of the institutional review board or ethics committee that approved the study<br>• Include the approval number and/or a statement indicating approval of this research<br>• Indicate the form of consent obtained (written/oral) or the reason that consent was not obtained (e.g. the data were analyzed anonymously)<br><br>**Animal Research (involving vertebrate animals, embryos or tissues)**<br>• Provide the name of the Institutional Animal Care and Use Committee (IACUC) or other relevant ethics board that reviewed the study protocol, and indicate whether they approved this research or granted a formal waiver of ethical approval<br>• Include an approval number if one was obtained<br>• If the study involved *non-human primates*, add *additional details* about animal welfare and steps taken to ameliorate suffering<br>• If anesthesia, euthanasia, or any kind of animal sacrifice is part of the study, include briefly which substances and/or methods were applied<br><br>**Field Research**<br><br>Include the following details if this study involves the collection of plant, animal, or other materials from a natural setting:<br>• Field permit number<br>• Name of the institution or relevant body that granted permission	
**Data Availability**<br><br>Authors are required to make all data underlying the findings described fully available, without restriction, and from the time of publication. PLOS allows rare exceptions to address legal and ethical concerns. See the PLOS Data Policy and FAQ for detailed information.	Yes - all data are fully available without restriction

A Data Availability Statement describing where the data can be found is required at submission. Your answers to this question constitute the Data Availability Statement and **will be published in the article**, if accepted.<br><br>**Important:** Stating 'data available on request from the author' is not sufficient. If your data are only available upon request, select 'No' for the first question and explain your exceptional situation in the text box.<br><br>Do the authors confirm that all data underlying the findings described in their manuscript are fully available without restriction?	
**Describe where the data may be found in full sentences. If you are copying our sample text, replace any instances of XXX with the appropriate details.**<br><br>• If the data are **held or will be held in a public repository**, include URLs, accession numbers or DOIs. If this information will only be available after acceptance, indicate this by ticking the box below. For example: *All XXX files are available from the XXX database (accession number(s) XXX, XXX.*).<br>• If the data are all contained **within the manuscript and/or Supporting Information files**, enter the following: *All relevant data are within the manuscript and its Supporting Information files.*<br>• If neither of these applies but you are able to provide **details of access elsewhere**, with or without limitations, please do so. For example:<br><br>*Data cannot be shared publicly because of [XXX]. Data are available from the XXX Institutional Data Access / Ethics Committee (contact via XXX) for researchers who meet the criteria for access to confidential data.*<br><br>*The data underlying the results presented in the study are available from (include the name of the third party*	*******@ PA @ Accept request data available on acceptance*********<br><br>All XXX files are available from the XXX database (accession number(s) XXX, XXX.).

and contact information or URL).<br>• This text is appropriate if the data are owned by a third party and authors do not have permission to share the data.<br><br>* typeset	
Additional data availability information:	Tick here if the URLs/accession numbers/DOIs will be available only after acceptance of the manuscript for publication so that we can ensure their inclusion before publication.

Powered by Editorial Manager® and ProduXion Manager® from Aries Systems Corporation

Manuscript
Click here to access/download;Manuscript;SWChars_Short_20230604_Plos_

Vocal signatures affected by population identity and environmental sound levels

Amber D. Fandel[1*], Kirsten Silva[1], and Helen Bailey[1]

[1]Chesapeake Biological Laboratory, University of Maryland Center for Environmental Science, 146 Williams St., Solomons, MD 20688, USA

[*]Corresponding author

E-mail: adfandel@gmail.com

Abstract

Passive acoustic monitoring has improved our understanding of vocalizing organisms in remote habitats and during all weather conditions. Many vocally active species are highly mobile, and their populations overlap. However, distinct vocalizations allow the tracking and discrimination of individuals or populations. Using signature whistles, the individually distinct calls of bottlenose dolphins, we calculated a minimum abundance of individuals, characterized and compared signature whistles from five locations, and determined re-occurrences of individuals throughout the Mid-Atlantic Bight and Chesapeake Bay, USA. We identified 1,888 signature whistles in which the duration, number of extrema, start, end, and minimum frequencies of signature whistles varied significantly by site. All characteristics of signature whistles were deemed important for determining from which site the whistle originated. With distinct characteristics and lack of spatial mixing, we suspect that Offshore dolphins are of a different population. Signature whistles were shorter when sound levels were higher. Using only the passively recorded vocalizations of this marine top predator, we obtained information about its population and how it is affected by ambient sound levels, which will increase as offshore wind energy is developed. In this rapidly developing area, these calls offer critical management insights for this protected species.

Introduction

In remote habitats and adverse weather conditions, visual monitoring of organisms is challenging. Under these conditions, passive acoustic monitoring is especially valuable for vocalizing species. Passive acoustic monitoring uses microphones (referred to as hydrophones in the underwater environment) to detect the sounds of vocalizing organisms. In the marine

environment, one of the most vocal groups of organisms are marine mammals, which produce a variety of calls. In addition to being able to detect their presence from these calls, some species have individual- (1) and pod-specific calls (2–4), allowing the detection of individual animals. The highly vocal and social bottlenose dolphin (*Tursiops truncatus*) produces individually distinct calls called signature whistles (5,6). These signature whistles comprise more than half of the whistles that wild bottlenose dolphins produce (7,8). They are formed in the first year of the dolphin's life (9) and remain relatively stable (5,9). These unique whistles have been used to both identify (1,10) and to estimate the number of individuals within small (< 100 individuals), resident (11–13) populations but less is known for larger, wide-ranging populations. In orcas (*Orcinus orca*), family pods are differentiated based on the characteristics of their vocalizations (3). While there has been some examination of dolphin vocalization differentiation (14,15), it is not yet known whether population-specific differences in bottlenose dolphin signature whistle characteristics.

The Atlantic coast of the USA is home to 19 different populations of bottlenose dolphins (16). The region along the coasts of Delaware, Maryland, and Virginia, within the southern Mid-Atlantic Bight, is one of the least studied areas for bottlenose dolphins on the east coast of the USA. This study aims to 1) determine a minimum estimate of the number of bottlenose dolphins in the Chesapeake Bay and waters of coastal Maryland, 2) whether there are site- or regionally-specific differences in signature whistle characteristics and how they relate to existing population boundaries, and 3) whether individual dolphins alter their signature whistles in response to ambient sound conditions. The findings from this study will help to determine bottlenose dolphin identities and populations from signature whistles. This has implications for population management in this urbanized waterway of the Chesapeake Bay and in the Mid-Atlantic Bight

where there offshore wind energy development has been proposed.

Methods

Signature whistles as a measure of minimum abundance

Passive acoustic monitoring occurred at five sites: two in the southern coastal Mid-Atlantic Bight (blue triangles; Sites 1, 2, Coastal region), two in the Chesapeake Bay (black circles; Site 3, 4, Bay region), and one offshore in the southern Mid-Atlantic Bight (red square; Site 5, Offshore region; Fig. 1). No permits were required for this work, as there was no handling or interaction with the study species, all monitoring was conducted by autonomous recorders. Sites 1, 2, and 5 were located 12, 31, and 64 km east of Ocean City, Maryland, USA, respectively (Fig. 1). Water depths at these sites ranged from approximately 20 – 42 m, and the acoustic recording instruments were deployed approximately 1 m above the ocean floor using bottom-anchored moorings (17).

Figure 1. Map of acoustic recording sites in the southern coastal Mid-Atlantic Bight, USA (blue triangles; Sites 1, 2), Chesapeake Bay (black circles; Site 3, 4), and offshore in the Mid-Atlantic Bight (red square; Site 5; following site nomenclature in Bailey et al. 2021 with additional sites). Sites at which data were gathered in previous studies are underlined. Basemap images are the intellectual property of ESRI and are reprinted from Esri under a CC BY license with permission from Esri and its licensors, all rights reserved. Credits: ESRI, HERE, Garmin, GEBCO, OpenStreetMap, the GIS user community, NOAA NGDC, and other contributors.

Details on acoustic data recording and identified signature whistles from 2016 through

2018 at Sites 1, 2, and 3 were described in (18). Previous recordings at Site 1 occurred between June and August in 2017 and July to October in 2018. At Site 2, recordings occurred between July and September of 2016, January to April and June to October in 2017, and June to December in 2018. Site 3 recordings occurred between June and August in 2018. Additional recordings for this study were obtained from Snap acoustic recorders (Loggerhead Instruments, FL, USA) deployed at Sites 4 and 5. At Site 4 in the Potomac River, the Snap was deployed in approximately 3 m of water from June to September 2019. At Site 5, 64 km from the Maryland coastline, the Snap was deployed from July to September 2018 in 42 m of water. At both sites, the Snap was approximately 1 m above the seabed sampling at 48 kHz with a sensitivity of 180.2 dB re 1V/µPa and a gain of 2.05 dB re 1µPa.

Due to longer deployments at Sites 4 and 5, , the Snap was duty-cycled for two minutes on and eight minutes off at Site 4 and one minute on and nine minutes off at Site 5. At Site 4, a subsample of days were analyzed because of the high rate of dolphin presence at this location. At Site 5 (as with Sites 1 and 2), the PAMGUARD Whistle and Moan Detector (19) was utilized to determine hours with possible dolphin presence and these hours were then manually searched for signature whistles with high signal-to-noise ratios. Signature whistles were obtained using the SIGID criteria (1)-the same whistle repeated in a pattern of two or more whistles (7) within 1–10 s of one another and with a minimum length of 0.2 s (20,21). Whistle contours (shape of the whistle) were obtained using Beluga software (https://synergy.st-andrews.ac.uk/soundanalysis) within MATLAB (Math-Works, Natick, Massachusetts, USA). Whistles with low signal-to-noise ratio or abundant non-linear features (22) that obscured the shape of the whistle could not be included in the analysis. Beluga contours were analyzed within the adaptive resonance theory neural network (ART-warp; vigilance threshold of 96%; 23) to identify when a whistle

reoccurred. Identical signature whistles were not considered re-occurrences if they occurred on the same day at the same site. A human analyst verified all whistle re-occurrences. (1,20). When the same signature whistle was identified at a different location or on a different day than the first instance, it was considered a re-occurrence of that individual signature whistle indicating the same dolphin had been detected again.

While other delphinid species are present in this region during the winter months, at the sites and times chosen, bottlenose dolphins were the most likely species present based on a previous analysis of visual sightings data in the study area (24,25). To confirm that the species detected were bottlenose, not common dolphin (*Delphinus delphis*) ROCCA, a species identification algorithm (26) was used. When comparing this algorithm to concurrent visual and passive acoustic observations in the Northwest Atlantic, ROCCA performed poorly (S. Van Parijs, personal communication). Similar poor results were obtained in our analyses. During the summer at the most inshore site (Site 1), bottlenose dolphins are the most likely species to be detected (25). In recordings from 21 hours on 16 days during the summer at Site 1, the ROCCA algorithm classified 61% of whistles as "Ambiguous" (n = 576), 13% as striped dolphin (*Stenella coeruleoalba*; n = 121), 11% as bottlenose dolphins (n = 103), 8% as common dolphins (*Delphinus delphis*, n = 74), 4% as Clymene dolphin (*Stenella clymene*, n = 39), 2 as Atlantic spotted dolphin (*Stenella frontalis*, n = 19), and 1% as Pantropical spotted dolphin (*Stenella attenuate*, n = 7). Clymene dolphins are typically found in waters deeper than 800 feet, and Pantropical spotted dolphins are not located in the Atlantic Ocean. Given the unreliability of these results, we were unable to confirm species identity using the acoustic data with ROCCA. Instead, we but relied on previous species assignments based on location and time of year from a random forest model using sighting data from a previous study (25).

Acoustic characteristics of signature whistles

To determine the acoustic characteristics of signature whistles from each site, we selected a subset of 100 signature whistles from each site (or the number of unique signature whistles identified if <100) whose full shape was clear. For each whistle, manual measurements were taken of the duration, start, end, maximum, minimum, and delta frequencies (maximum minus minimum frequency), and number of local extrema (e.g. local minima and maxima) including the start and end of the whistle (similar to (12,27); Fig 2). The distribution of the signature whistles' characteristics were analyzed for normality using Shapiro-Wilk tests, and Box-Cox analyses (MASS package in R; 28) were used to determine whether transformations of the characteristics were necessary for parametric analyses.

Figure 2. Diagram of whistle showing characteristics measured including start, end, minimum, maximum, and delta frequencies, number of extrema, and duration.

A multivariate analysis of variance (MANOVA) in R was utilized to determine whether the whistle characteristics (response variables) were related to the site (Sites 1-5) or region (Bay, Coastal, Offshore) at which the signature whistle was detected (explanatory variables). Post-hoc individual pairwise t-tests with Bonferroni adjusted p-values were used to investigate which and how signature whistle characteristics varied between sites and regions. The Boruta package in R (29) utilizes random forest models to assess factor importance in determining an identity (e.g. site or region) and was employed to determine the relative importance of each signature whistle characteristic in that process. The maximum number of runs for the Boruta model was 10,000.

Signature whistle variation in relation to ambient sound levels

To understand whether bottlenose dolphins alter their signature whistles in response to ambient sound levels, a subset of 100 clear signature whistles that reoccurred at least once, were selected for measurement (duration, start, end, maximum, minimum, and delta frequencies, number of local extrema). Ambient sound levels were calculated in MATLAB (MathWorks, Natick, Massachusetts, USA) as the relative broadband (up to 24 kHz, given the sampling rate of 48 kHz) sound pressure level (SPL; dB re 1µPa root-mean-square (rms)) during the recording in which the signature whistle occurred (two or five minutes in duration). Sound levels were also calculated for the duration of each acoustic deployment to determine overall ambient sound levels. Generalized estimating equations-generalized linear models (GEEs) in R (geepack package in R; 30) were used to determine which signature whistles characteristics were affected by changes in ambient sound levels. The identity of the signature whistle was treated as a cluster and an exchangeable correlation structure was used in the GEEs.

Results

Signature whistles as an indicator of minimum abundance

A total of 1,172 unique signature whistles were detected at coastal Site 1, 327 at coastal Site 2, and 19 at Site 3 in the Bay (18). A total of 333 unique signature whistles were detected at Site 4 in the Potomac River in the Chesapeake Bay, and 37 at the offshore site, Site 5 (Table 1). An individuals' dolphin's presence is indicated by the presence of its signature whistle, so these

counts of unique signature whistles represent a minimum abundance of bottlenose dolphins occurring at each site.

Table 1. For each site and region, the recording period, total number of signature whistles detected, and total number and percentage of signature whistles selected for acoustic characteristic measurement.

Site	Region	Recording period	Hours analyzed	Total number of unique SW	Number of SWs with measured characteristics	Percentage of total SW with measured characteristics (%)
1	Coastal	June-Aug 2017, July-Oct 2018	268	1172	100	9
2	Coastal	July-Sept 2016, Jan-April, June-Oct 2017, June-Dec 2018	521	327	100	31
3	Bay	May-Sept 2018	2488	19	19	100
4	Bay	June-Sep 2019	311	333	100	33
5	Offshore	July- Sep 2018	415	37	37	100

Acoustic characteristics of signature whistles

For the subset of signature whistles for which whistle characteristics were measured Table 1), the distribution of these characteristics (start, end, maximum, minimum, and delta frequencies, duration, number of local extrema) were non-parametric (Shapiro-Wilk test; $p < 0.05$). Following Box-Cox analyses and appropriate transformations, the data were tested again for normality, but only the start, minimum, and delta frequencies became normally distributed. Because the distribution of end frequencies remained non-parametric after transformation, a square root (sqrt) transformation was utilized for consistency with other frequency measures. Only transformed values of signature whistle characteristics were used in the subsequent analyses of these whistles.

A MANOVA indicated that start ($F_{4,352} = 2.71$, $p = 0.03$), end ($F_{4,352} = 4.52$, $p = 0.01$), and minimum frequencies ($F_{4,352} = 10.3$, $p < 0.01$) as well as duration ($F_{4,352} = 9.36$, $p < 0.01$) and number of extrema ($F_{4,352} = 7.28$, $p < 0.01$) of signature whistles varied significantly by site. Pairwise t-tests indicated that the start and end frequencies of signature whistles were higher (Fig. 3a, b) and delta frequencies were lower (Fig. 3d) at Site 5 compared to Site 4. Minimum frequencies of signature whistles at Site 5 were higher than all other sites (Fig. 3c), and signature whistles from Site 2 had significantly longer durations than whistles from Site 1, 3, and 4 (Fig. 3e). The number of extrema were significantly fewer in signature whistles from Site 5 compared to any site other than Site 3, and there were significantly more local extrema at Site 1 compared to Site 2 (Fig. 3f).

Figure 3. Standard boxplot of the a) square root (sqrt) of the start frequency, b) sqrt of the end frequency, c) sqrt of the minimum frequency, d) sqrt of the delta frequency, e) log of the duration, and f) log of the number of extrema for signature whistles at each site. Asterisks indicate an adjusted p-value (Bonferonni method) of less than 0.05 in pairwise t-tests. Blue boxes indicate sites in the Coastal region, black are sites in the Chesapeake Bay, and red is the site in the Offshore region.

A MANOVA also indicated that all characteristics of the signature whistles except the delta frequencies varied significantly by region. Offshore signature whistles had significantly higher start, end, and minimum frequencies compared to those from the Coastal and Bay regions (Fig. 4a, b, c). The duration of signature whistles from the Bay were shorter than those from the Coastal or Offshore regions (Fig. 4d), and Offshore signature whistles had significantly fewer extrema than the Bay or Coastal whistles (Fig. 4e).

Figure 4. Standard boxplot of the a) square root (sqrt) of the start frequency, b) sqrt of the end frequency c) sqrt of the minimum frequency, d) log of the duration, and e) log of the number of extrema for signature whistles in each region. Asterisks indicate an adjusted p-value of less than 0.05 (Bonferonni method) in pairwise t-tests.

All signature whistle characteristics were deemed important for determining site identity, but minimum frequency and duration were the most important factors (Fig. 5a). The duration, minimum, maximum, end, and delta frequencies were the important characteristics in determining the region identity of signature whistles (Fig. 5b). Consideration of the start

frequency and number of extrema decreased the performance of the model when determining the region to which a signature whistle belonged (Fig 5b).

Figure 5. Results of the Boruta random forest model indicating the ranked importance of signature whistle characteristics in determining a whistle's (a) site and (b) regional identities. Green boxes indicate important characteristics, red indicates characteristics that decreased the performance of the model, and the blue are shadow factors used to test the model. Shadow factors are shuffled duplicate copies of factors added to remove correlations between variables.

Median daily sound levels in all sites and regions were significantly different from one another ($p < 0.01$). Site 1 was loudest followed by Site 5, Site 2, Site 3, and Site 4 (Fig. 6). The Offshore region had the second highest daily median ambient sound levels (Fig. 6) and the highest median daily minimum sound levels (127 dB re 1µPa, sd = 0.2 dB). Site 4 had the largest range in sound levels (Fig. 6).

Figure 6. Median daily sound levels (SPL rms: dB re 1µPa) at each site (a) and region (b). Asterisks indicate an adjusted p-value (Bonferonni method) of less than 0.05 in the pairwise t-tests.

Signature whistle variation in relation to ambient sound levels

In total, 201 unique signature whistles reoccurred a total of 252 times at the five different sites (Fig. 7). Of the 19 unique signature whistles at Site 3, 26% (n = 5) reoccurred at Site 4 (Fig. 7). Of the 333 unique signature whistles identified at Site 4, 24 unique signature whistles

reoccurred 27 times within the site. Of the 37 unique signature whistles detected at Site 5, three signature whistles reoccurred within the site. There were three re-occurrences between Site 5 and Site 2 (two of the same whistle; 5% of all unique signature whistles from Site 5) and one at Site 1 (3%; Fig. 7).

Figure 7. Network diagram showing signature whistle reo-ccurrences (gray lines) between sites (circles labeled with site number) within the study area. Blue nodes indicate sites in the Coastal region, black indicate sites in the Chesapeake Bay, and red indicates a site in the Offshore region. Size of the node indicates the number of signature whistles detected at each site.

The random selection of 100 unique signature whistles that were analyzed to determine how signature whistle characteristics varied at differing sound levels included 230 total whistle occurrences: 132 whistles at Site 1, 49 at Site 2, 9 at Site 3, 36 at Site 4, and 4 at Site 5. When changes in signature whistle characteristics were modeled in relation to the ambient sound levels during which they occurred, only the duration of the signature whistles significantly varied in relation to changing ambient sound levels, decreasing as sound levels increased (Estimate = -0.005, S.E. = 0.003, Wald = 4.08, $p = 0.04$). All other signature whistle characteristics did not vary between re-occurrences when sound levels varied.

Discussion

We determined that the characteristics of bottlenose dolphin signature whistles significantly varied by site and region, which may represent acoustic differences amongst the signature whistles of estuarine, coastal, and offshore bottlenose dolphin populations. Notably, the

same individual adjusted the duration of its signature whistle in relation to the ambient sound level when it was emitted. This is the first quantitative investigation into the effects of location and ambient sound levels on individually identifiable dolphin calls.

Characterizing signature whistles may allow for the acoustic differentiation of populations or stocks. This is especially valuable in areas of population overlap such as the Mid-Atlantic Bight and the Chesapeake Bay. Our study area is within the home range of up to four different populations of bottlenose dolphins; the Western North Atlantic (WNA) Northern Migratory Coastal, WNA Southern Migratory Coastal, Northern North Carolina Estuarine System, and the WNA Offshore stocks. The numerous differences in the acoustic characteristics of signature whistles from bottlenose dolphins in the Offshore region (Site 5, 42 m depth) and lack of re-occurrences of these whistles in the Chesapeake Bay suggests that these dolphins may be from the WNA Offshore stock. The movement patterns and number of individuals in this offshore population are poorly understood. Signature whistle analysis may allow us to track individuals over time and space, which is especially valuable in this area where visual methods of tracking are challenging, and offshore renewable energy is being developed.

Large numbers of bottlenose dolphins seasonally inhabit the Chesapeake Bay (31,32), and acoustic niche partitioning (33,34) in signature whistles may be occurring (15,35) due to population overlap. However, it is still unclear to which population(s) these bottlenose dolphins in the Bay belong. Population-level variation in vocal calls (i.e. vocal dialects) have been detected in multiple species (3,36–38) and are likely utilized in this highly vocal and mobile species. Some signature whistles, but not all, detected in the Chesapeake Bay (Sites 3 and 4) also occurred in the Coastal region (Sites 1 and 2), suggesting that the Chesapeake Bay may be a mixing ground for multiple populations including the WNA Northern Migratory Coastal, WNA

Southern Migratory Coastal, and Northern North Carolina Estuarine System stocks. It also indicates that the ranges of these populations may be more extensive than previously thought and re-consideration of stock delineations may be necessary.

The importance of identifying the stock identity of dolphins in the Chesapeake Bay is further necessitated by the evidence of calves (32). To understand to which stock the calves may belong, the study area could be expanded to the north and to the south, extending into the known ranges of other populations of this species. This would offer the opportunity to record signature whistles from individuals that have been photo-identified. The feasibility of using signature whistles and their characteristics for stock assignment could then be determined, allowing a better understanding of stock structure, which is vital for management and consideration during environmental impact assessments.

Additionally, the importance of signature whistles in communicating an individual's identity may necessitate its acoustic differentiation from other whistle types. Signature whistles at Site 2 were generally longer (1.13 s), had lower minimum (5966 Hz), higher maximum (12750 Hz), and larger delta frequencies (6784 Hz) than a random selection of whistles from the same site (0.40 s, 6792 Hz, 100075 Hz, 3282 Hz; 27). This dissimilarity of characteristics between a random selection of whistles and the signature whistles from Site 2 suggests that there are some acoustic properties of signature whistles (in addition to their repeated pattern) that indicates their identity as signature whistles.

High ambient sound levels (> 120 dB re 1µPa) were recorded in this study area, and the duration of dolphin signature whistles decreased as ambient sound levels increased. Because our calculation of ambient sound levels did not exclude whistles, higher sound levels could also be indicative of more whistles within recordings. Additionally, because sound with frequencies

above 24 kHz could not be detected in our recordings, higher frequency signature whistles would have been missed in our analyses (39). While an increase in maximum frequency in response to higher sound levels was found in some studies (27,40,41), the maximum frequency of some signature whistles in this region were high(9) and at the upper limit of our sampling frequency. Thus, alternative acoustic compensation strategies such as shortening the duration of signature whistles may have been necessary (42–46). These compensation strategies, however, may not be adequate to avoid signal masking (47).

Offshore, where median daily ambient sound levels were highest, signature whistles were higher frequency (start, end, minimum) and simpler (with fewer extrema). Whistles with higher frequencies (27,41) and fewer extrema (27,40) are common where low frequency ambient sound levels are higher. Large vessels and high sea states are the primary contributors to consistently high ambient sound levels at this location near the vessel separation schema leading into the busy Port of Wilmington, Delaware. Dolphins of this region are likely utilizing higher frequency whistles to avoid acoustic masking from vessel traffic (48,49).

This study found that the characteristics of bottlenose dolphin signature whistles differed significantly between sites and regions. Understanding how signature whistles vary by location provides insight into the movements of individual bottlenose dolphins and how this may delineate the distribution of specific populations in the region (50). In addition, bottlenose dolphins adjusted the duration of their signature whistles in relation to the ambient sound environment in which they were emitted, possibly to compensate for the increased noise and reduce the likelihood of interruption of their call. Increasing sound levels in the ocean may continue to cause changes in dolphin calls leading to information loss (51) and reducing cohesion amongst dolphin groups, including mother-calf pairs, who use signature whistles to facilitate

reunions (8). Sound levels in this region will rise due to development of offshore wind energy, coastal development, bridge construction, and increasing vessel traffic. It is particularly important to understand how individuals from particular populations may be exposed to sound from various human activities and hence how they may be impacted by masking of calls and potential disruption to their communication that could affect their fitness. The use of signature whistles to track individuals can also aid in determining how long individuals take to return to an area and resume normal behaviors after exposure to elevated sound levels. Such information will be vital for management of this region to determine the potential effects of, responses to, and recovery from anthropogenic sound pollution (50).

Acknowledgements

Thank you to Elizabeth Gryzb, Elizabeth McDonald, and Aimee Hoover at the Chesapeake Biological Laboratory for their initial analyses, Matt Ogburn at the Smithsonian Environmental Research Center for assistance with obtaining recordings, and Aaron Rice at Cornell University.

References

1. Janik VM, Sayigh LS. Communication in bottlenose dolphins: 50 years of signature whistle research. J Comp Physiol. 2013 Jun;199(6):479–89.
2. Filatova OA, Miller PJOO, Yurk H, Samarra FIPP, Hoyt E, Ford JKBB, et al. Killer whale call frequency is similar across the oceans, but varies across sympatric ecotypes. J Acoust Soc Am. 2015 Jul;138(1):251–7.
3. Ford JKB, Fisher HD. Killer whale (Orcinus orca) dialects as an indicator of stocks in British Columbia. 1982.
4. Scheer M, Hoffman B, Behr PI. Discrete pod-specific call repertoires among short-finned pilot whales (Globicephala macrorhynchus) off the SW coast of Tenerife, Canary Islands. In: World Marine Mammal Science Conference, 20-24 January,

Monaco by European Cetacean Society and Society for Marine Mammalogy. Monaco; 1998. p. 1998.

5. Sayigh LS, Tyack PL, Wells RS, Scott MD. Signature whistles of free-ranging bottlenose dolphins Tursiops truncatus: stability and mother-offspring comparisons. Behav Ecol Sociobiol. 1990;26(4):247–60.
6. Caldwell MC, Caldwell DK. Individualized Whistle Contours in Bottle-nosed Dolphins (Tursiops truncatus). Nature. 1965;207(4995):434–5.
7. Cook MLH, Sayigh LS, Blum JE, Wells RS. Signature-whistle production in undisturbed free-ranging bottlenose dolphins (Tursiops truncatus). Proceedings of the Royal Society B: Biological Sciences. 2004 May 22;271(1543):1043–9.
8. Smolker RA, Mann J, Smuts BB. Use of signature whistles during separations and reunions by wild bottlenose dolphin mothers and infants. Behav Ecol Sociobiol. 1993;33(6):393–402.
9. Caldwell MC, Caldwell DK, Tyack PL. Review of the signature-whistle hypothesis for the Atlantic bottlenose dolphin. In: Leatherwood S, Reeves RR, editors. The bottlenose dolphin. New York, NY: Academic Press; 1990. p. 199–234.
10. Quick NJ, Janik VM. Bottlenose dolphins exchange signature whistles when meeting at sea. Proceedings of the Royal Society B: Biological Sciences. 2012;279(1738):2539–45.
11. Erbe C, Salgado-Kent C, de Winter S, Marley S, Ward R. Matching Signature Whistles with Photo-Identification of Indo-Pacific Bottlenose Dolphins (Tursiops aduncus) in the Fremantle Inner Harbour, Western Australia. Acoust Aust. 2020 Apr 1;48(1):23–38.
12. Kriesell HJ, Elwen SH, Nastasi A, Gridley T. Identification and characteristics of signature whistles in wild bottlenose dolphins (Tursiops truncatus) From Namibia. PLoS One. 2014;9(9).
13. Longden EG, Elwen SH, McGovern B, James BS, Embling CB, Gridley T. Mark-recapture of individually distinctive calls - A case study with signature whistles of bottlenose dolphins (Tursiops truncatus). J Mammal. 2020;101(5):1289–301.
14. Morisaka T, Shinohara M, Nakahara F, Akamatsu T, Morisaka T, Shinohara M, et al. Geographic variations in the whistles among three Indo-Pacific bottlenose dolphin. Fisheries Science. 2005;71:568–76.
15. Campbell G. Quantitative comparison of bottlenose dolphin (Tursiops truncatus) whistles from three geographic regions. 2004.
16. Hayes SA, Josephson E, Maze-Foley K, Rosel PE, Byrd B, Chavez-Rosales S, et al. US Atlantic and Gulf of Mexico Marine Mammal Stock Assessments [Internet]. NOAA Technical Memorandum. Woods Hole, MA; 2021. Available from: https://media.fisheries.noaa.gov/2021-07/Atlantic 2020 SARs Final.pdf?null%09
17. Garrod A, Fandel AD, Wingfield JE, Fouda L, Rice AN, Bailey H. Validating automated click detector dolphin detection rates and investigating factors affecting performance. J Acoust Soc Am. 2018 Aug;144(2):931–9.
18. Bailey H, Fandel AD, Silva KK, Gryzb E, McDonald E, Hoover AL, et al. Identifying and predicting occurrence and abundance of a vocal animal species based on individually specific calls. Ecosphere. 2021;12(8).
19. Gillespie D, White P, Caillat M, Gordon J. Development and Implementation of Automatic Classification of Odontocetes within PAMGUARD. 2011.

20. Janik VM, King SL, Sayigh LS, Wells RS. Identifying signature whistles from recordings of groups of unrestrained bottlenose dolphins (Tursiops truncatus). Mar Mamm Sci. 2013 Jan;29(January):109–22.
21. Gridley T, Cockcroft VG, Hawkins ER, Blewitt ML, Morisaka T, Janik VM. Signature whistles in free-ranging populations of Indo-Pacific bottlenose dolphins, Tursiops aduncus. Mar Mamm Sci. 2014;30(2):512–27.
22. Sportelli JJ, Jones BL, Ridgway SH. Non-linear phenomena: a common acoustic feature of bottlenose dolphin (Tursiops truncatus) signature whistles. Bioacoustics. 2022;
23. Deecke VB, Janik VM. Automated categorization of bioacoustic signals: Avoiding perceptual pitfalls. J Acoust Soc Am. 2006 Jan;119(1):645–53.
24. Barco S, Burt L, Deperte A, Digiovanni R. Prepared for the Maryland Department of Natural Resources Marine Mammal and Sea Turtle Sightings in the Vicinity of the Maryland Wind Energy Area All images-Riverhead Foundation for Marine Research and Preservation, Permit # 15575. 2013.
25. Bailey H, Lyubchich V, Wingfield JE, Fandel AD, Garrod A, Rice AN. Empirical evidence that large marine predator foraging behavior is consistent with area-restricted search theory. Ecology. 2019 Aug 1;100(8):1–9.
26. Oswald JN, Rankin S, Barlow JP, Lammers MO. A tool for real-time acoustic species identification of delphinid whistles. J Acoust Soc Am. 2007 Jul;122(1):587–95.
27. Fouda L, Wingfield JE, Fandel AD, Garrod A, Hodge KB, Rice AN, et al. Dolphins simplify their vocal calls in response to increased ambient noise. Biol Lett. 2018 Oct 1;14(10).
28. Venables WN, Ripley BD. Modern Applied Statistics with S. Fourth. New York, NY: Springer; 2002.
29. Kursa MB, Rudnicki WR. Feature selection with the boruta package. J Stat Softw. 2010;36(11):1–13.
30. Højsgaard S, Halekoh U. The R Package geepack for Generalized Estimating Equations. J Stat Softw. 2006;(2006):1–6.
31. Rodriguez LK, Fandel AD, Colbert BR, Testa JC, Bailey H. Spatial and temporal variation in the occurrence of bottlenose dolphins in the Chesapeake Bay, USA, using citizen science sighting data. PLoS One. 2021;16(5):e0251637.
32. Barco SG, Swingle WMark, McLellan WA, Harris RN, Pabst DAnn. Local abundance and distribution of bottlenose dolphins (Tursiops truncatus) in the nearshore water of Virginia Beach, Virginia. Mar Mamm Sci. 1999;15(2).
33. Hart PJ, Paxton K, Ibanez T, Tredinnick G, Sebastián-González E, Tanimoto-Johnson A. Acoustic niche partitioning in two tropical wet forest bird communities. bioRxiv. 2020;2020.08.17.253674.
34. Krause B, Farina A. Using ecoacoustic methods to survey the impacts of climate change on biodiversity. BIOC. 2016;195:245–54.
35. Luís AR, May-Collado LJ, Rako-Gospić N, Gridley T, Papale E, Azevedo A, et al. Vocal universals and geographic variations in the acoustic repertoire of the common bottlenose dolphin. Sci Rep. 2021;11(1):1–9.
36. Astrom G, Stolt B. Regional song dialects of the Ortolan Bunting Emberiza hortulana L. in Sweden. Ornis Svec. 1993;3:1–10.

37. Barker AJ, Veviurko G, Bennett NC, Hart DW, Mograby L, Lewin GR. Cultural transmission of vocal dialect in the naked mole-rat. Science (1979). 2021;371(6528):503–7.
38. Marler P, Tamura M. Song " Dialects " in Three Populations of White-Crowned Sparrows. Condor. 1962;64(5):368–77.
39. Daisy Kaplan J, Reiss D. Whistle acoustic parameters in atlantic spotted dolphins (Stenella frontalis) and bottlenose dolphins (Tursiops truncatus) in two locations in the bahamas and comparisons with other populations. Aquat Mamm. 2017;43(4):364–77.
40. Marley SA, Salgado Kent CP, Erbe C, Parnum IM. Effects of vessel traffic and underwater noise on the movement, behaviour and vocalisations of bottlenose dolphins in an urbanised estuary. Sci Rep. 2017 Dec 1;7(1).
41. van Ginkel C, Becker DM, Gowans S, Simard P. Whistling in a noisy ocean: Bottlenose dolphins adjust whistle frequencies in response to real-time ambient noise levels. Bioacoustics. 2018 Aug 24;27(4):391–405.
42. Picciulin M, Sebastianutto L, Codarin A, Calcagno G, Ferrero EA. Brown meagre vocalization rate increases during repetitive boat noise exposures : A possible case of vocal compensation. Journal of the Acoustical Society of America. 2012;132.
43. Hotchkin CF, Parks SE, Weiss DJ. Vocal modifications in primates: Effects of noise and behavioral context on vocalization structure. Proceedings of Meetings on Acoustics. 2013;19(010061).
44. Fournet MEH, Silvestri M, Clark CW, Klinck H, Rice AN. Limited vocal compensation for elevated ambient noise in bearded seals : implications for an industrializing Arctic Ocean. Proc Biol Sci. 2021;288.
45. Miksis-Olds JL, Tyack PL. Manatee (Trichechus manatus) vocalization usage in relation to environmental noise levels. Journal of the Acoustical Society of America. 2009;125:1806–15.
46. Krahforst CS, Sprague MW, Luczkovich JJ. The impact of vessel noise on oyster toadfish (Opsanus tau) communication. Proceedings of Meetings on Acoustics. 2016;27(010031).
47. Templeton CN, Zollinger SA, Brumm H. Traffic noise drowns out great tit alarm calls. Current Biology. 2016;26(22):R1173–4.
48. May-Collado LJ, Wartzok D. A Comparison of Bottlenose Dolphin Whistles in the Atlantic Ocean: Factors Promoting Whistle Variation. J Mammal. 2008;89(5):1229–40.
49. Ansmann IC, Goold JC, Evans PGH, Simmonds M, Keith SG. Variation in the whistle characteristics of short-beaked common dolphins, Delphinus delphis, at two locations around the British Isles. Journal of Marine Biological Assessment, UK. 2007;87:19–26.
50. Weilgart LS. The impacts of anthropogenic ocean noise on cetaceans and implications for management. Can J Zool. 2007;85(11):1091–116.
51. Marten K, Quine D, Marler P. Sound Transmission and Its Significance for Animal Vocalization: II. Tropical Forest Habitats [Internet]. Vol. 2, Sociobiology. 1977. Available from: https://about.jstor.org/terms

Figure 1

Click here to access/download;Figure;SWCharsMap(2).pdf

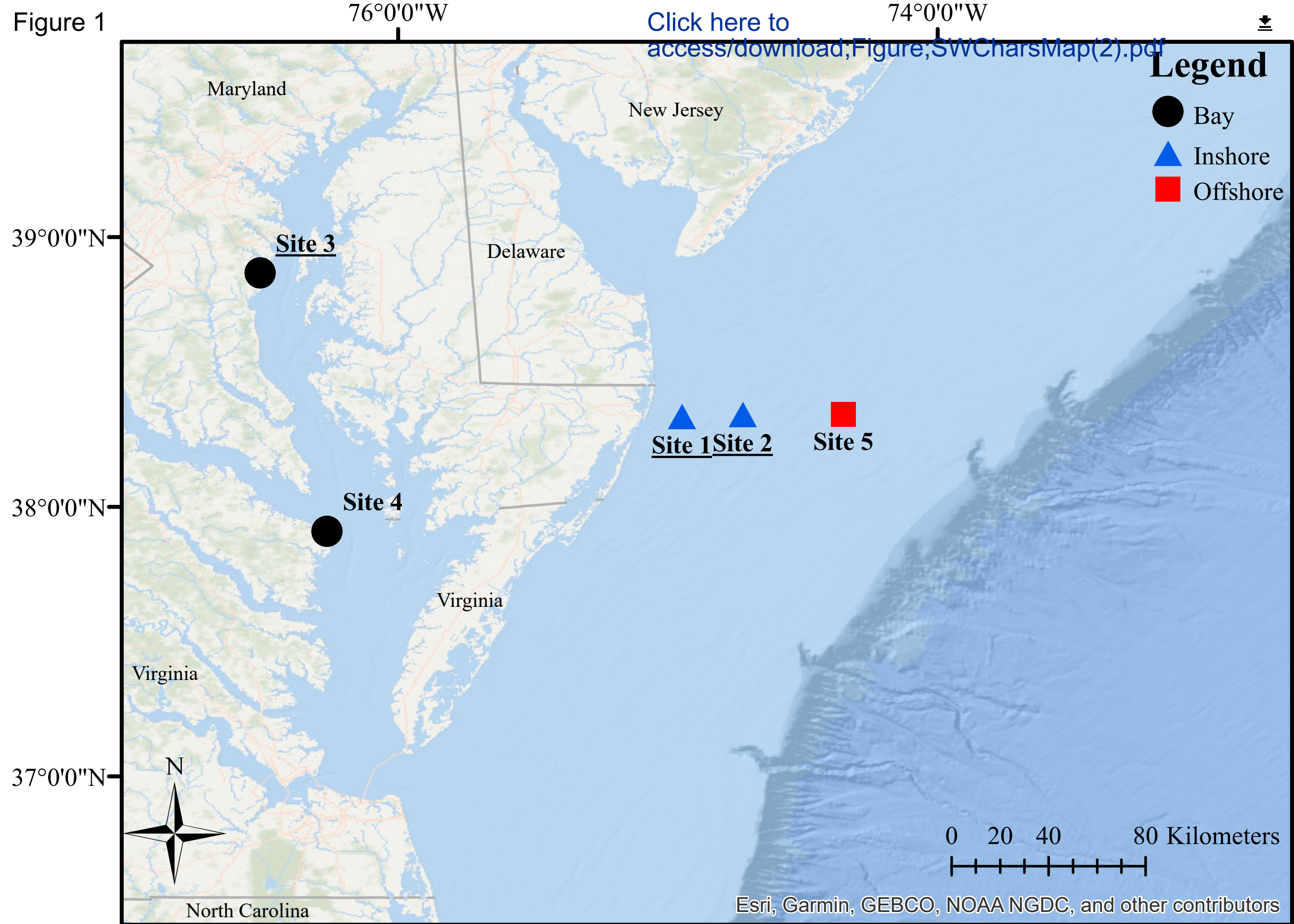

Figure 2

Click here to access/download;Figure;DolphinWhistleCharImage_Fig2.pdf

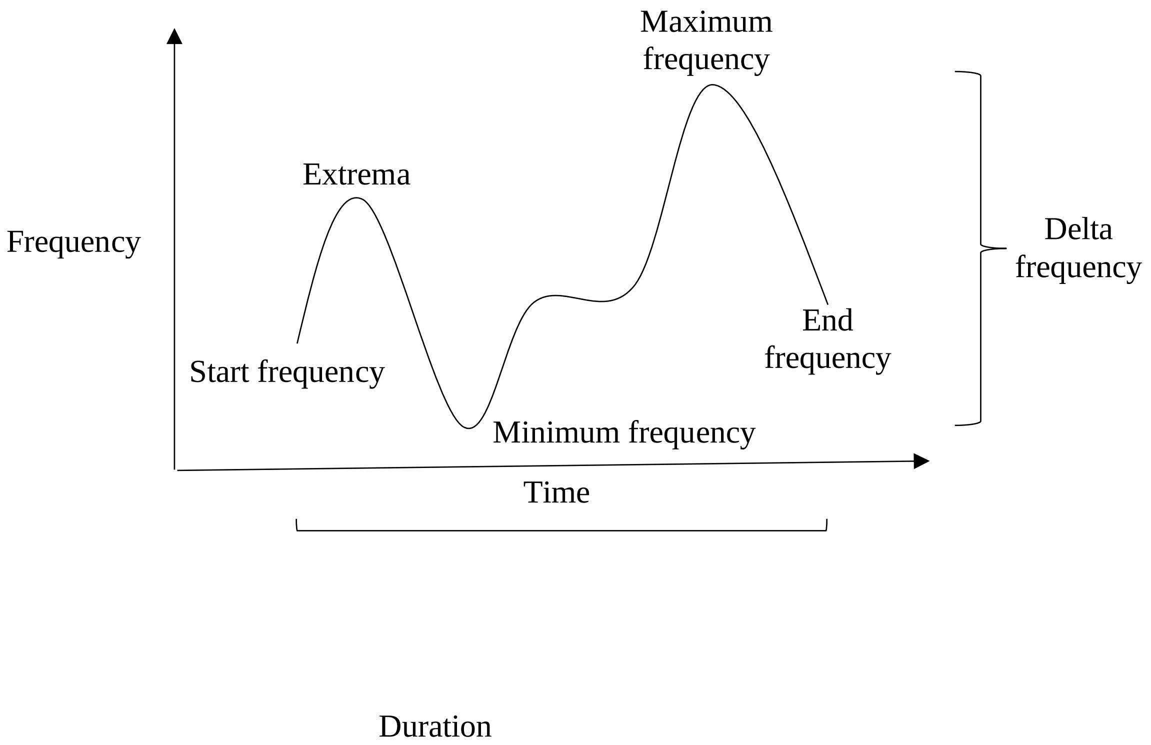

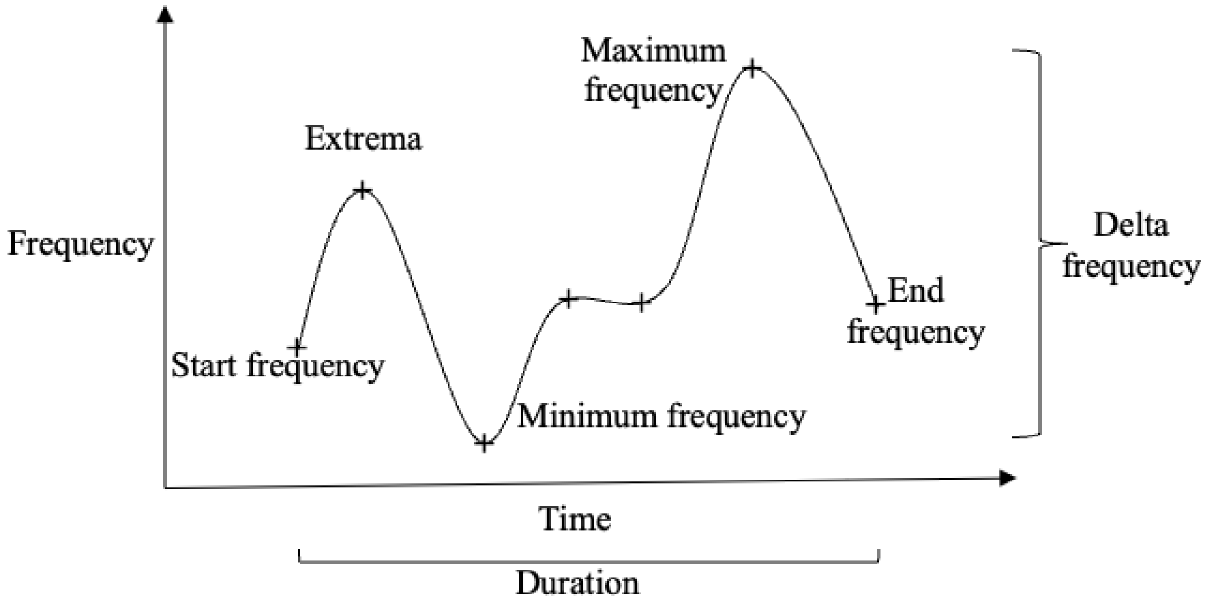

Figure 3

Click here to access/download;Figure;Fig3.pdf

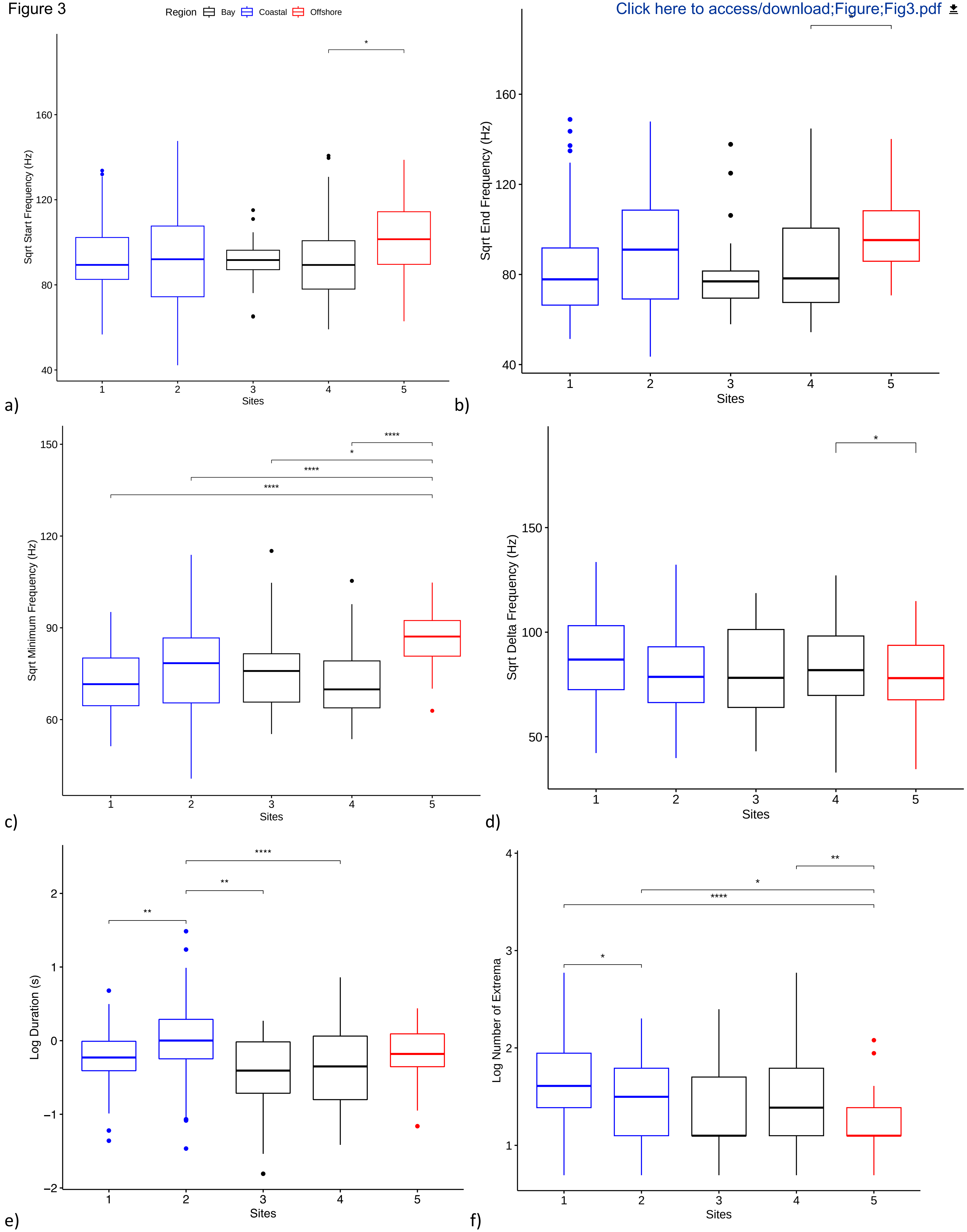

Figure 4

Click here to access/download;Figure;Fig4.pdf

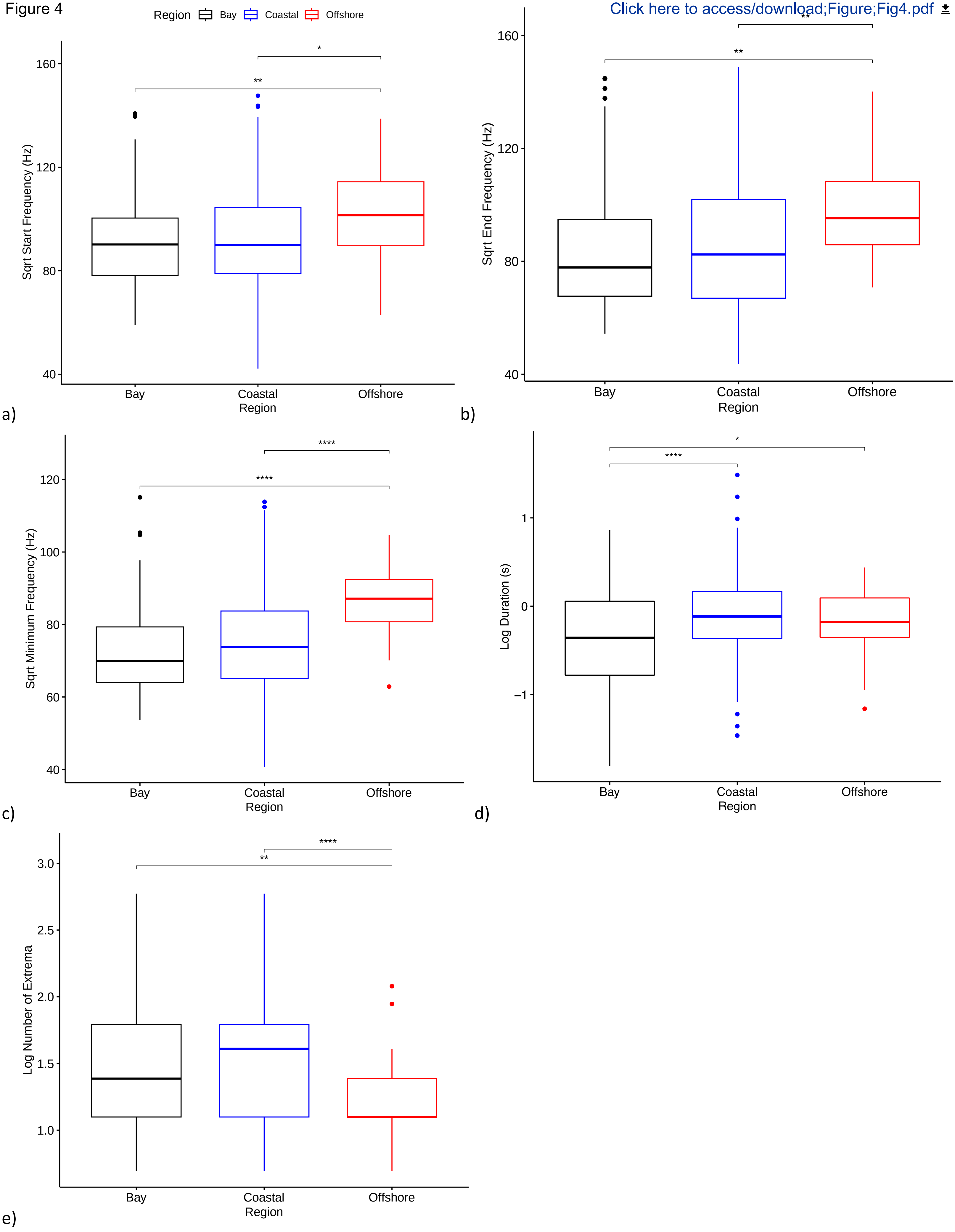

Figure 5

Click here to access/download;Figure;Fig5.pdf

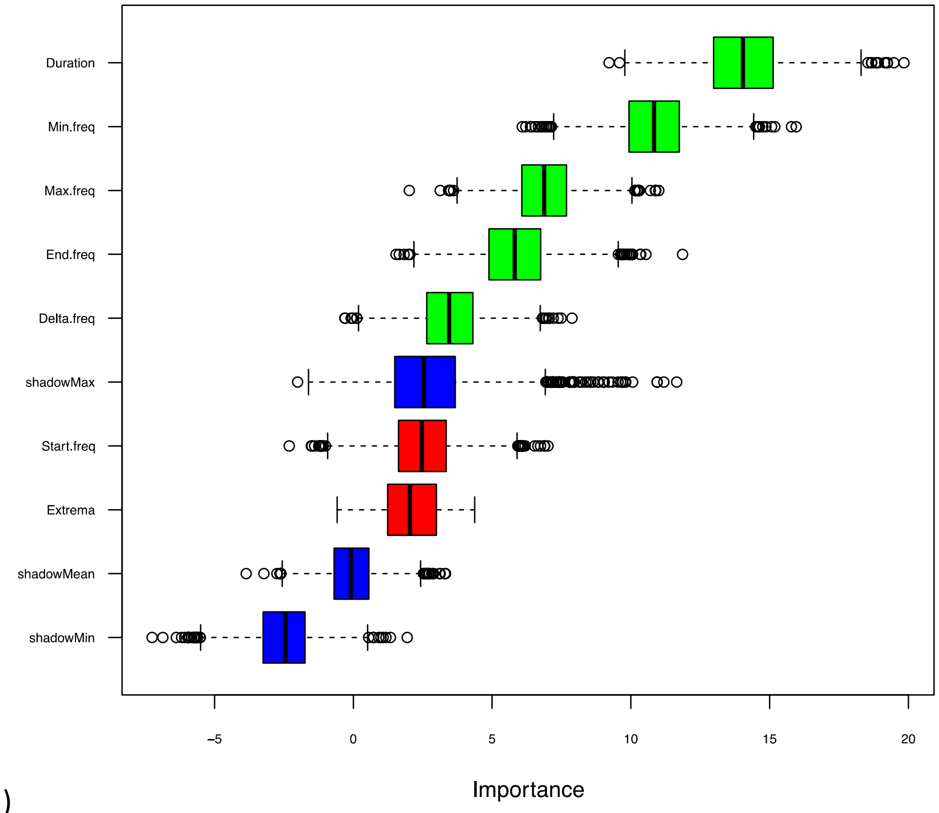

a)

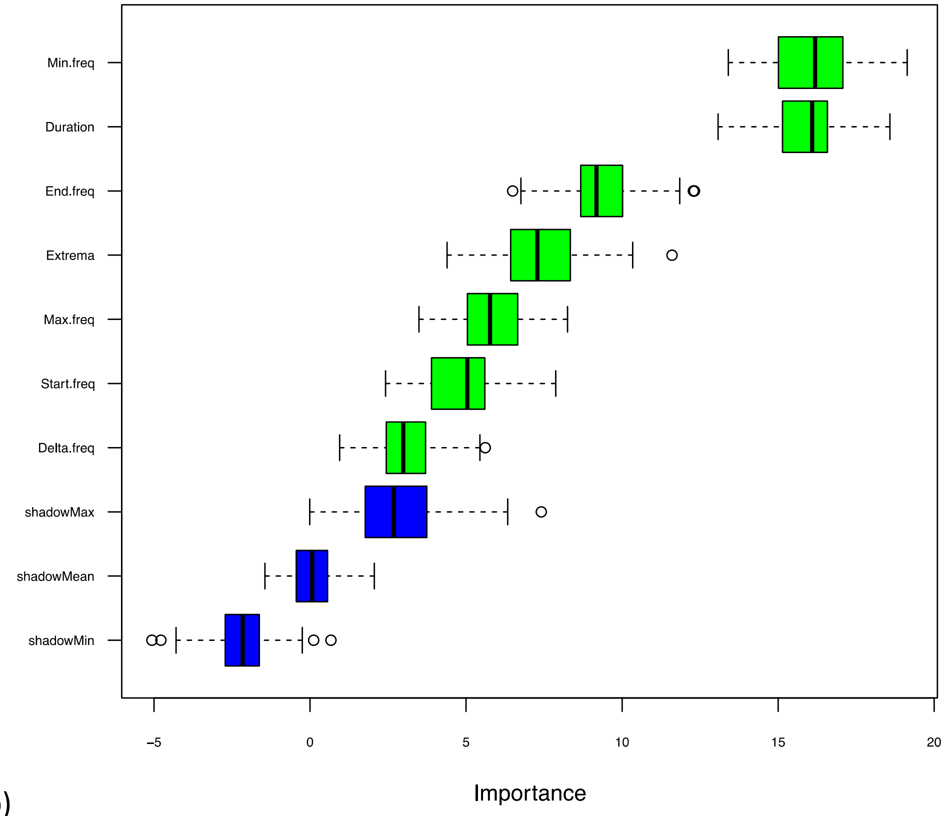

b)

Figure 6

Click here to access/download;Figure;Fig6.pdf

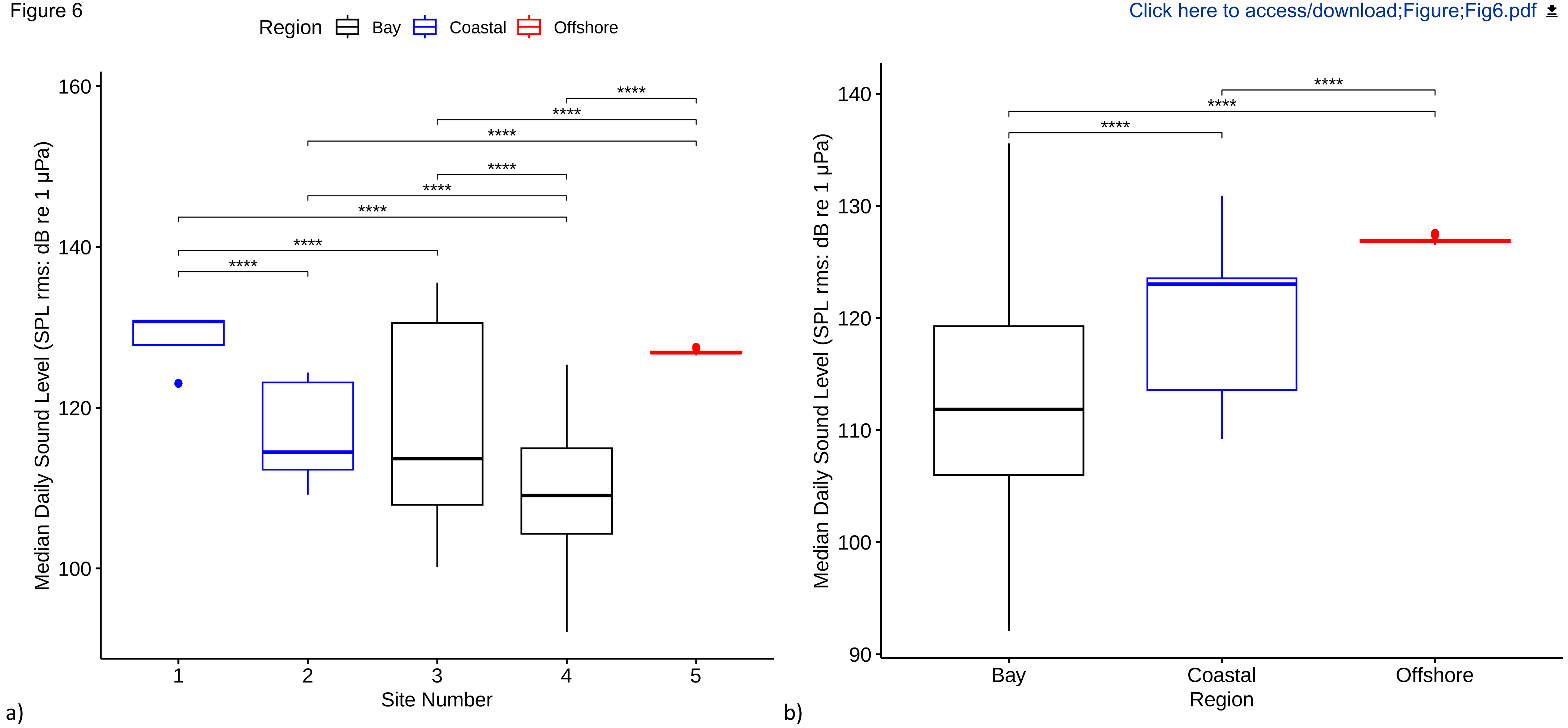

Figure 7

Click here to access/download;Figure;Slide7.png

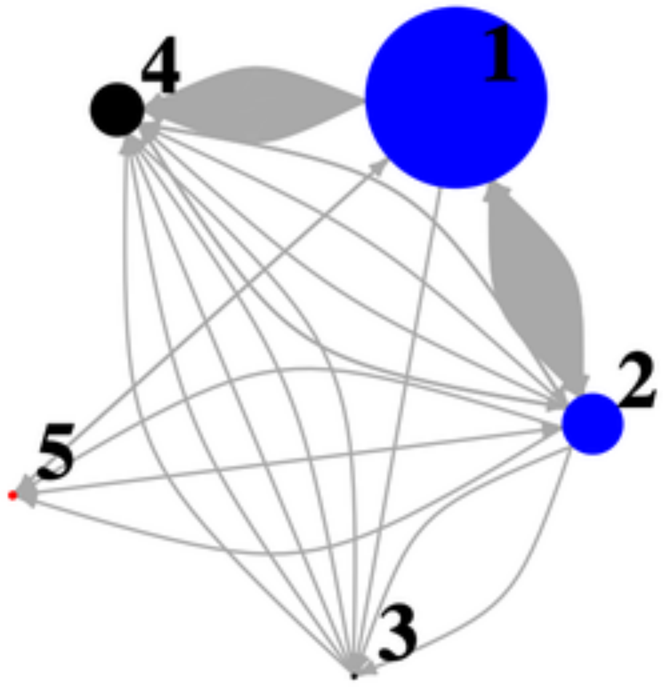

Revised Manuscript with Track Changes

Vocal signatures affected by population identity and environmental sound levels

Amber D. Fandel[1*], Kirsten Silva[1], and Helen Bailey[1]

[1]Chesapeake Biological Laboratory, University of Maryland Center for Environmental Science, 146 Williams St., Solomons, MD 20688, USA

[*]Corresponding author

E-mail: adfandel@gmail.com

Abstract

Passive acoustic monitoring has improved our understanding of vocalizing organisms in remote habitats and during all weather conditions. Many vocally active species are highly mobile, and their populations overlap. However, distinct vocalizations allow the tracking and discrimination of individuals or populations. Using signature whistles, the individually distinct calls of bottlenose dolphins, we calculated a minimum abundance of individuals, characterized and compared signature whistles from five locations, and determined re-occurrences of individuals throughout the Mid-Atlantic Bight and Chesapeake Bay, USA. We identified 1,888 signature whistles in which the duration, number of extrema, start, end, and minimum frequencies of signature whistles varied significantly by site. All characteristics of signature whistles were deemed important for determining from which site the whistle originated. With distinct characteristics and lack of spatial mixing, we suspect that Offshore dolphins are of a different population. Signature whistles were shorter when sound levels were higher. Using only the passively recorded vocalizations of this marine top predator, we obtained information about its population and how it is affected by ambient sound levels, which will increase as offshore wind energy is developed. In this rapidly developing area, these calls offer critical management insights for this protected species.

Introduction

In remote habitats and ~~challenging~~ adverse weather conditions, visual monitoring of organisms is challenging. Under~~In~~ these conditions, passive acoustic monitoring is especially valuable for vocalizing species. Passive acoustic monitoring uses microphones (referred to as hydrophones in the underwater environment) to detect the sounds of vocalizing organisms. In the

marine environment, one of the most~~A highly~~ vocal groups of organisms are ~~of species in the marine environment is~~ marine mammals, which produce a variety of calls. In addition to being able to detect~~ing~~ their presence from these~~ir~~ calls, some species have individual- (1) and pod-specific calls (2–4), allowing ~~detection~~ the detection of individual animals. The highly vocal and social bottlenose dolphin (*Tursiops truncatus*) produces individually distinct calls called signature whistles (5,6). These signature whistles comprise more than half of the whistles that wild bottlenose dolphins produce (7,8). They are formed in the first year of the dolphin's life (9) and remain relatively stable (5,9). These unique whistles have been used to both identify (1,10) and to estimate the number of individuals within small (< 100 individuals), resident (11–13) populations but ~~have not yet been tested on~~less is known for larger, ~~free~~wide-ranging populations. In orcas (*Orcinus orca*), family pods are differentiated based on the characteristics of their vocalizations (3). While there has been some examination of dolphin vocalization differentiation ~~has been examined~~ (14,15), it is not yet known whether population-specific differences ~~occur~~ in bottlenose dolphin~~'s~~ signature whistle characteristics.

The Atlantic coast of the USA is home to 19 different populations of bottlenose dolphins (16). The region along the coasts of Delaware, Maryland, and Virginia, ~~or~~ within the southern Mid-Atlantic Bight, is one of the least studied areas for bottlenose dolphins on the east coast of the USA. This study aims to 1) determine a minimum estimate of the number of bottlenose dolphins in the Chesapeake Bay and waters of coastal Maryland, 2) whether there are site- or regionally-specific differences in signature whistle characteristics and how they relate to existing population boundaries, and 3) whether individual dolphins alter their signature whistles in response to ambient sound conditions. The findings from this study will help to determine bottlenose dolphin identities and populations from signature whistles. This has implications for

population management in this urbanized waterway of the Chesapeake Bay and in the Mid-Atlantic Bight where there ~~an area proposed for~~ offshore wind energy development has been proposed.

Methods

Signature whistles as a measure of minimum abundance

Passive acoustic monitoring occurred at five sites: two in the southern coastal Mid-Atlantic Bight (blue triangles; Sites 1, 2, Coastal region), two in the Chesapeake Bay (black circles; Site 3, 4, Bay region), and one offshore in the southern Mid-Atlantic Bight (red square; Site 5, Offshore region; Fig. 1). No permits were required for this work, as there was no handling or interaction with the study species, all monitoring was conducted by autonomous recorders. Sites 1, 2, and 5 were located 12, 31, and 64 km east of Ocean City, Maryland, USA, respectively (Fig. 1). Water depths at these sites ranged from approximately 20 – 42 m, and the acoustic recording instruments were deployed approximately 1 m above the ocean floor using bottom-anchored moorings (17).

Figure 1. Map of acoustic recording sites in the southern coastal Mid-Atlantic Bight, USA (blue triangles; Sites 1, 2), Chesapeake Bay (black circles; Site 3, 4), and offshore in the Mid-Atlantic Bight (red square; Site 5; following site nomenclature in Bailey et al. 2021 with additional sites). Sites at which data were gathered in previous studies are underlined. Basemap images are the intellectual property of ESRI and are reprinted from Esri under a CC BY license with permission

from Esri and its licensors, all rights reserved. Credits: ESRI, HERE, Garmin, GEBCO, OpenStreetMap, the GIS user community, NOAA NGDC, and other contributors.

Details on acoustic data recording and identified signature whistles from 2016 through 2018 at Sites 1, 2, and 3 were described in (18). Previous recordings at Site 1 occurred between June and August in 2017 and July to October in 2018. At Site 2, recordings occurred between July and September of 2016, January to April and June to October in 2017, and June to December in 2018. Site 3 recordings occurred between June and August in 2018. Additional recordings for this study were obtained from Snap acoustic recorders (Loggerhead Instruments, FL, USA) ~~were~~ deployed at Sites 4 and 5. At Site 4 in the Potomac River, the Snap was deployed in approximately 3 m of water from June to September 2019. At Site 5, 64 km from the Maryland coastline, the Snap was deployed from July to September 2018 in 42 m of water. At both sites, the Snap was approximately 1 m above the seabed sampling at 48 kHz with a sensitivity of 180.2 dB re 1V/µPa and a gain of 2.05 dB re 1µPa.

Due to longer deployments at Sites 4 and 5, ~~To increase the time span of the recordings~~, the Snap was duty-cycled for two minutes on and eight minutes off at Site 4 and one minute on and nine minutes off at Site 5. A~~A~~t Site 4, a subsample of days were analyzed because of the high rate of dolphin presence at this location~~recordings were investigated for signature whistles only on days during which a visual sighting occurred~~. At Site 5 (as with Sites 1 and 2), the PAMGUARD Whistle and Moan Detector (19) was utilized to determine hours with possible dolphin presence and these hours were then manually searched for signature whistles with high signal-to-noise ratios. Signature whistles were obtained using the SIGID criteria (1)~~-~~the same whistle repeated in a pattern of two or more whistles (7) within 1–10 s of one another and with a

Formatted: Indent: First line: 0"

Formatted: Font: 12 pt

Formatted: Font: 12 pt

Formatted: Font: (Default) Times New Roman, 12 pt

Formatted: Font: 12 pt

minimum length of 0.2 s (20,21). Whistle contours (shape of the whistle) were obtained using Beluga software (https://synergy.st-andrews.ac.uk/soundanalysis) within MATLAB (Math-Works, Natick, Massachusetts, USA). Whistles with low signal-to-noise ratio or abundant non-linear features (22) that obscured the shape of the whistle could not be included in the analysis. Beluga contours were analyzed within the adaptive resonance theory neural network (ART-warp; vigilance threshold of 96%; (23)) to identify when a whistle reoccurred. Identical signature whistles were not considered re-occurrences if they occurred on the same day at the same site. A human analyst verified all whistle re-occurrences. ~~The SIGID criteria~~ (1,20) ~~and methodology from 14 (ARTWARP with manual validation) were utilized to identify high signal-to-noise ratio signature whistles and determine when they reoccurred.~~ When the same signature whistle was identified at a different location or on a different day than the first instance, it was considered a re-occurrence of that individual signature whistle indicating the same dolphin had been detected again. ~~Signature whistles were considered reoccurrences when they occurred on different days or in different locations.~~

While other delphinid species are present in this region during the winter months, at the sites and times chosen, bottlenose dolphins were the most likely species present based on a previous analysis of visual sightings data in the study area (24,25). To confirm that the species detected were bottlenose, not common dolphin (*Delphinus delphis*) ROCCA, a species identification algorithm (26) was used. When comparing this algorithm to concurrent visual and passive acoustic observations in the Northwest Atlantic, ROCCA performed poorly (S. Van Parijs, personal communication). Similar poor results were obtained in our analyses. During the summer at the most inshore site (Site 1), bottlenose dolphins are the most likely species to be detected (25). In recordings from 21 hours on 16 days during the summer at Site 1, the ROCCA

Formatted: Font: 12 pt

Formatted: Font: 12 pt

Formatted: Font: 12 pt

Formatted: Font: 12 pt

Formatted: Font: 12 pt

Formatted: Font: 12 pt

Formatted: Font: 12 pt

Formatted: Font: 12 pt

Formatted: Font: 12 pt

Formatted: Font: 10 pt, Pattern: Clear (White)

algorithm classified 61% of whistles as “Ambiguous” (n = 576), 13% as striped dolphin (*Stenella coeruleoalba*; n = 121), 11% as bottlenose dolphins (n = 103), 8% as common dolphins (*Delphinus delphis*, n = 74), 4% as Clymene dolphin (*Stenella clymene*, n = 39), 2 as Atlantic spotted dolphin (*Stenella frontalis*, n = 19), and 1% as Pantropical spotted dolphin (*Stenella attenuate*, n.= 7). Clymene dolphins are typically found in waters deeper than 800 feet, and Pantropical spotted dolphins are not located in the Atlantic Ocean. Given the unreliability of these results, ~~Thus,~~ we were unable to confirm species identity using the acoustic datas ~~and~~ with ROCCA. Instead, we but relied on previous species assignments based on location and time of year from a ~~ly tested~~ random forest model using sighting data from a previous studys ~~using environmental and location data to confirm species identity~~ (25).

Acoustic characteristics of signature whistles

To determine the acoustic characteristics of signature whistles from each site, we selected a subset of 100 signature whistles from each site (or the number of unique signature whistles identified if <100) whose full shape was clear. For each whistle, manual measurements were taken of the duration, start, end, maximum, minimum, and delta frequencies (maximum minus minimum frequency), and number of local extrema (e.g. local minima and maxima) including the start and end of the whistle (similar to (12,27); Fig 2). The distribution of the signature whistles’ characteristics were analyzed for normality using Shapiro-Wilk tests, and Box-Cox analyses (MASS package in R; (28) were used to determine whether transformations of the characteristics were necessary for parametric analyses.

Figure 2. Diagram of whistle showing characteristics measured including start, end, minimum, maximum, and delta frequencies, number of extrema, and duration.

A multivariate analysis of variance (MANOVA) in R was utilized to determine whether the whistle characteristics (response variables) were related to the site (Sites 1-5) or region (Bay, Coastal, Offshore) at which the signature whistle was detected (explanatory variables). Post-hoc individual pairwise t-tests with Bonferroni adjusted p-values were used to investigate which and how signature whistle characteristics varied between sites and regions. The Boruta package in R (29) utilizes random forest models to assess factor importance in determining an identity (e.g. site or region) and was employed to determine the relative importance of each signature whistle characteristic in that process. The maximum number of runs for the Boruta model was 10,000.

Signature whistle variation in relation to ambient sound levels

To understand whether bottlenose dolphins alter their signature whistles in response to ambient sound levels, a subset of 100 clear signature whistles that reoccurred at least once, either at the same or at a different site, were selected for acoustic characteristic measurement (duration, start, end, maximum, minimum, and delta frequencies, number of local extrema). Ambient sound levels were calculated in MATLAB (MathWorks, Natick, Massachusetts, USA) as the relative broadband (up to 24 kHz, given the sampling rate of 48 kHz) sound pressure level (SPL; dB re 1µPa root-mean-square (rms)) during the recording in which the signature whistle occurred. (two or five minutes in duration). Sound levels were also calculated for the duration of each acoustic deployment to determine overall ambient sound levels. Generalized estimating equations-generalized linear models (GEEs) in R (geepack package in R; (30) were used to determine

which signature whistles characteristics were affected by changes in ambient sound levels. The identity of the signature whistle was treated as a cluster and an exchangeable correlation structure was used in the GEEs. ~~Ambient sound levels were calculated as the broadband (up to 24 kHz) sound pressure level (SPL; dB re 1μPa root-mean-square (rms)) during the recording in which the signature whistle occurred.~~

Results

Signature whistles as an indicator of minimum abundance

A~~Previous research detected a~~ total of 1,172 unique signature whistles were detected at coastal Site 1, 327 at coastal Site 2, and 19 at Site 3 in the Bay (18). A total of 333 unique signature whistles were detected at Site 4 in the Potomac River in the Chesapeake Bay, and 37 at the offshore site, Site 5 (Table 1). An individuals' dolphin's presence is indicated by the presence of its signature whistle, so t~~T~~hese counts of unique signature whistles represent a minimum abundance of bottlenose dolphins occurring at each ~~at these~~ site~~s~~.

Table 1. For each site and region, the recording period, total number of signature whistles detected, and total number and percentage of signature whistles selected for acoustic characteristic measurement.

Formatted: Font: Bold

Site	Region	Recording period	Hours analyzed	Total number of	Number of SWs with	Percentage of total SW with measured characteristics (%)

				unique SW	measured characteristics	
1	Coastal	June-Aug 2017, July-Oct 2018	268	1172	100	9
2	Coastal	July-Sept 2016, Jan-April, June-Oct 2017, June-Dec 2018	521	327	100	31
3	Bay	May-Sept 2018	2488	19	19	100
4	Bay	June-Sep 2019	311	333	100	33
5	Offshore	July- Sep 2018	415	37	37	100

Acoustic characteristics of signature whistles

For the subset of signature whistles for which whistle characteristics were measured Table 1), t~~T~~he distribution of these ~~signature whistle~~ characteristics (start, end, maximum, minimum, and delta frequencies, duration, number of local extrema) were non-parametric (Shapiro-Wilk test; $p < 0.05$). Following Box-Cox analyses and appropriate transformations, the data were tested again for normality, but only the start, minimum, and delta frequencies became normally distributed. Because the distribution of end frequencies remained non-parametric after transformation, a square root (sqrt) transformation was utilized for consistency with other frequency measures. Only transformed values of signature whistle characteristics were used in the subsequent analyses of these whistles.

A MANOVA indicated that start ($F_{4,352}$ = 2.71, p = 0.03), end ($F_{4,352}$ = 4.52, p = 0.01), and minimum frequencies ($F_{4,352}$ = 10.3, p < 0.01) as well as duration ($F_{4,352}$ = 9.36, p < 0.01) and number of extrema ($F_{4,352}$ = 7.28, p < 0.01) of signature whistles varied significantly by site. Pairwise t-tests indicated that the start and end frequencies of signature whistles were higher (Fig. 3a, b) and delta frequencies were lower (Fig. 3d) at Site 5 compared to Site 4. Minimum frequencies of signature whistles at Site 5 were higher than all other sites (Fig. 3c), and signature whistles from Site 2 had significantly longer durations than whistles from Site 1, 3, and 4 (Fig. 3e). The number of extrema were significantly fewer in signature whistles from Site 5 compared to any site other than Site 3, and there were significantly more local extrema at Site 1 compared to Site 2 (Fig. 3f).

Figure 3. Standard boxplot of the a) square root (sqrt) of the start frequency, b) sqrt of the end frequency, c) sqrt of the minimum frequency, d) sqrt of the delta frequency, e) log of the duration, and f) log of the number of extrema for signature whistles at each site. Asterisks indicate an adjusted p-value (Bonferonni method) of less than 0.05 in pairwise t-tests. Blue boxes indicate sites in the Coastal region, black are sites in the Chesapeake Bay, and red is the site in the Offshore region.

A MANOVA also indicated that all characteristics of the signature whistles except the delta frequencies varied significantly by region. Offshore signature whistles had significantly higher start, end, and minimum frequencies compared to those from the Coastal and Bay regions (Fig. 4a, b, c). The duration of signature whistles from the Bay were shorter than those from the

Coastal or Offshore regions (Fig. 4d), and Offshore signature whistles had significantly fewer extrema than the Bay or Coastal whistles (Fig. 4e).

Figure 4. Standard boxplot of the a) square root (sqrt) of the start frequency, b) sqrt of the end frequency c) sqrt of the minimum frequency, d) log of the duration, and e) log of the number of extrema for signature whistles in each region. Asterisks indicate an adjusted p-value of less than 0.05 (Bonferonni method) in pairwise t-tests.

All signature whistle characteristics were deemed important for determining site identity, but minimum frequency and duration were the most important factors (Fig. 5a). The duration, minimum, maximum, end, and delta frequencies were the important characteristics in determining the region identity of signature whistles (Fig. 5b). Consideration of the start frequency and number of extrema decreased the performance of the model when determining the region to which a signature whistle belonged (Fig 5b).

Figure 5. Results of the Boruta random forest model indicating the ranked importance of signature whistle characteristics in determining a whistle's (a) site and (b) regional identities. Green boxes indicate important characteristics, red indicates characteristics that decreased the performance of the model, and the blue are shadow factors used to test the model. Shadow factors are shuffled duplicate copies of factors added to remove correlations between variables.

Median daily sound levels in all sites and regions were significantly different from one another ($p < 0.01$). Site 1 was loudest followed by Site 5, Site 2, Site 3, and Site 4 (Fig. 6). The

Offshore region had the second highest daily median ambient sound levels (Fig. 6) and the highest median daily minimum sound levels (127 dB re 1µPa, sd= 0.2 dB). Site 4 had the largest range in sound levels (Fig. 6).

Figure 6. Median daily sound levels (SPL rms: dB re 1µPa) at each site (a) and region (b). Asterisks indicate an adjusted p-value (Bonferonni method) of less than 0.05 in the pairwise t-tests.

Signature whistle variation in relation to ambient sound levels

In total, 201 unique signature whistles reoccurred a total of 252 times at the five different sites (Fig. 7). Of the 19 unique signature whistles at Site 3, 26% (n = 5) reoccurred at Site 4 (Fig. 7). Of the 333 unique signature whistles identified at Site 4, 24 unique signature whistles reoccurred 27 times within the site. Of the 37 unique signature whistles detected at Site 5, three signature whistles reoccurred within the site. There were three re-occurrences between Site 5 and Site 2 (two of the same whistle; 5% of all unique signature whistles from Site 5) and one at Site 1 (3%; Fig. 7).

Figure 7. Network diagram showing signature whistle reo-ccurrences (gray lines) between sites (circles labeled with site number) within the study area. Blue nodes indicate sites in the Coastal region, black indicate sites in the Chesapeake Bay, and red indicates a site in the Offshore region. Size of the node indicates the number of signature whistles detected at each site.

The random selection of 100 unique signature whistles that were analyzed to determine how signature whistle characteristics varied at differing sound levels included 230 total whistle occurrences: 132 whistles at Site 1, 49 at Site 2, 9 at Site 3, 36 at Site 4, and 4 at Site 5. When changes in signature whistle characteristics were modeled in relation to the ambient sound levels during which they occurred, only t~~T~~he duration of the signature whistles significantly varied in relation to changing ambient sound levels, decreasing as sound levels increased (Estimate = -0.005, S.E. = 0.003, Wald = 4.08, p = 0.04). All other signature whistle characteristics did not vary between re-occurrences when sound levels varied.

Discussion

We determined that the characteristics of bottlenose dolphin signature whistles significantly varied by site and region, which may represent acoustic differences amongst the signature whistles of estuarine, coastal, and offshore bottlenose dolphin populations. Notably, the same individual adjusted the duration of its signature whistle in relation to the ambient sound level when it was emitted. This is the first quantitative investigation into the effects of location and ambient sound levels on individually identifiable dolphin calls.

Characterizing signature whistles may allow for the acoustic differentiation of populations or stocks. This is especially valuable in areas of population overlap such as the Mid-Atlantic Bight and the Chesapeake Bay. Our study area is within the home range of up to four different populations of bottlenose dolphins; the Western North Atlantic (WNA) Northern Migratory Coastal, WNA Southern Migratory Coastal, Northern North Carolina Estuarine System, and the WNA Offshore stocks. The numerous differences in the acoustic characteristics of signature whistles from bottlenose dolphins in the Offshore region (Site 5, 42 m depth) and

lack of re-occurrences of these whistles in the Chesapeake Bay suggests that these dolphins may be from the WNA Offshore stock. The movement patterns and number of individuals in this offshore population are poorly understood. Signature whistle analysis may allow us to track individuals over time and space, which is especially valuable in this area where visual methods of tracking are challenging, and offshore renewable energy is being developed.

Large numbers of bottlenose dolphins seasonally inhabit the Chesapeake Bay (31,32), and acoustic niche partitioning (33,34) in signature whistles may be occurring (15,35) due to population overlap. However, it is still unclear to which population(s) these bottlenose dolphins in the Bay belong. Population-level variation in vocal calls (i.e. vocal dialects) have been detected in multiple species (3,36–38) and are likely utilized in this highly vocal and mobile species. Some signature whistles, but not all, detected in the Chesapeake Bay (Sites 3 and 4) also occurred in the Coastal region (Sites 1 and 2), suggesting that the Chesapeake Bay may be a mixing ground for multiple populations including the WNA Northern Migratory Coastal, WNA Southern Migratory Coastal, and Northern North Carolina Estuarine System stocks. It also indicates that the ranges of these populations may be more extensive than previously thought and re-consideration of stock delineations may be necessary.

The importance of identifying the stock identity of dolphins in the Chesapeake Bay is further necessitated by the evidence of calves (32). To understand to which stock the calves may belong, the study area could be expanded to the north and to the south, extending into the known ranges of other populations of this species. This would offer the opportunity to record signature whistles from individuals that have been photo-identified. The feasibility of using signature whistles and their characteristics for stock assignment could then be determined, allowing a

better understanding of stock structure, which is vital for management and consideration during environmental impact assessments.

Additionally, the importance of signature whistles in communicating an individual's identity may necessitate its acoustic differentiation from other whistle types. Signature whistles at Site 2 were generally longer (1.13 s), had lower minimum (5966 Hz), higher maximum (12750 Hz), and larger delta frequencies (6784 Hz) than a random selection of whistles from the same site (0.40 s, 6792 Hz, 100075 Hz, 3282 Hz; (27). This dissimilarity of characteristics between a random selection of whistles and the signature whistles from Site 2 suggests that there are some acoustic properties of signature whistles (in addition to their repeated pattern) that indicates their identity as signature whistles.

High ambient sound levels (> 120 dB re 1µPa) were recorded in this study area, and the duration of dolphin signature whistles decreased as ambient sound levels increased. Because our calculation of ambient sound levels did not exclude whistles, higher sound levels could also be indicative of more whistles within recordings. Additionally, because sound with frequencies above 24 kHz could not be detected in our recordings, higher frequency signature whistles would have been missed in our analyses (39). While aAn increase in maximum frequency in response to higher sound levels was found in some studies (27,40,41), but the maximum frequency of some signature whistles in this region were near the highest recorded frequency for a whistle (9) and at the upper limit of our sampling frequency. Thus, alternative acoustic compensation strategies such as shortening the duration of signature whistles may have been necessary (42–46). These compensation strategies, however, may not be adequate to avoid signal masking (47).

Offshore, where median daily ambient sound levels were highest, signature whistles were higher frequency (start, end, minimum) and simpler (with fewer extrema). Whistles with higher

frequencies (27,41) and fewer extrema (27,40) are common where low frequency ambient sound levels are higher. Large vessels and high sea states are the primary contributors to consistently high ambient sound levels at this location near the vessel separation schema leading into the busy Port of Wilmington, Delaware. Dolphins of this region are likely utilizing higher frequency whistles to avoid acoustic masking from vessel traffic (48,49).

This study found that the characteristics of bottlenose dolphin signature whistles differed significantly between sites and regions. Understanding how signature whistles vary by location provides insight into the movements of individual bottlenose dolphins and how this may delineate the distribution of specific populations in the region (50). In addition, bottlenose dolphins adjusted the duration of their signature whistles in relation to the ambient sound environment in which they were emitted, possibly as a way to compensate for the increased noise and~~to~~ reduce the ~~possibility~~ likelihood of interruption of their call. Increasing sound levels in the ocean may continue to cause changes in dolphin calls leading to information loss (51) and reducing cohesion amongst dolphin groups, including mother-calf pairs, who use signature whistles to facilitate reunions (8). Sound levels in this region will rise due to development of offshore wind energy, coastal development, bridge construction, and increasing vessel traffic. It is particularly important to understand how individuals from particular populations may be exposed to sound from various human activities and hence how they may be impacted by masking of calls and potential disruption to their communication that could affect their fitness. The use of signature whistles to track individuals can also aid in determining how long individuals take to return to an area and resume normal behaviors after exposure to elevated sound levels. Such information ~~These findings help us to understand how the changing ocean is affecting this critical communication and~~ will be vital for management of this region to

determine the potential effects of, responses to, and recovery from anthropogenic sound pollution (50)~~, which is subject to rising~~. ~~sound levels due to offshore wind development, coastal development, bridge construction, and increasing vessel traffic.~~

Acknowledgements~~ and funding~~

Thank you to Elizabeth Gryzb, Elizabeth McDonald, and Aimee Hoover at the Chesapeake Biological Laboratory for their initial analyses, Matt Ogburn at the Smithsonian Environmental Research Center for assistance with obtaining recordings, and Aaron Rice at Cornell University. ~~The Maryland Department of Natural Resources secured funding for this project from the Maryland Energy Administration's Offshore Wind Development Fund (Contract number 14-18-2420 MEA). Amber Fandel received support from Maryland Sea Grant under award NA22OAR4170020 from the National Oceanic and Atmospheric Administration, U.S. Department of Commerce. The views and conclusions contained in this document are those of the authors and should not be interpreted as representing the opinions or policies of the Maryland Department of Natural Resources, or the Maryland Energy Administration. Mention of trade names or commercial products does not constitute their endorsement by the state.~~

References

1. Janik VM, Sayigh LS. Communication in bottlenose dolphins: 50 years of signature whistle research. J Comp Physiol. 2013 Jun;199(6):479–89.
2. Filatova OA, Miller PJOO, Yurk H, Samarra FIPP, Hoyt E, Ford JKBB, et al. Killer whale call frequency is similar across the oceans, but varies across sympatric ecotypes. J Acoust Soc Am. 2015 Jul;138(1):251–7.

3. Ford JKB, Fisher HD. Killer whale (Orcinus orca) dialects as an indicator of stocks in British Columbia. 1982.
4. Scheer M, Hoffman B, Behr PI. Discrete pod-specific call repertoires among short-finned pilot whales (Globicephala macrorhynchus) off the SW coast of Tenerife, Canary Islands. In: World Marine Mammal Science Conference, 20-24 January, Monaco by European Cetacean Society and Society for Marine Mammalogy. Monaco; 1998. p. 1998.
5. Sayigh LS, Tyack PL, Wells RS, Scott MD. Signature whistles of free-ranging bottlenose dolphins Tursiops truncatus: stability and mother-offspring comparisons. Behav Ecol Sociobiol. 1990;26(4):247–60.
6. Caldwell MC, Caldwell DK. Individualized Whistle Contours in Bottle-nosed Dolphins (Tursiops truncatus). Nature. 1965;207(4995):434–5.
7. Cook MLH, Sayigh LS, Blum JE, Wells RS. Signature-whistle production in undisturbed free-ranging bottlenose dolphins (Tursiops truncatus). Proceedings of the Royal Society B: Biological Sciences. 2004 May 22;271(1543):1043–9.
8. Smolker RA, Mann J, Smuts BB. Use of signature whistles during separations and reunions by wild bottlenose dolphin mothers and infants. Behav Ecol Sociobiol. 1993;33(6):393–402.
9. Caldwell MC, Caldwell DK, Tyack PL. Review of the signature-whistle hypothesis for the Atlantic bottlenose dolphin. In: Leatherwood S, Reeves RR, editors. The bottlenose dolphin. New York, NY: Academic Press; 1990. p. 199–234.
10. Quick NJ, Janik VM. Bottlenose dolphins exchange signature whistles when meeting at sea. Proceedings of the Royal Society B: Biological Sciences. 2012;279(1738):2539–45.
11. Erbe C, Salgado-Kent C, de Winter S, Marley S, Ward R. Matching Signature Whistles with Photo-Identification of Indo-Pacific Bottlenose Dolphins (Tursiops aduncus) in the Fremantle Inner Harbour, Western Australia. Acoust Aust. 2020 Apr 1;48(1):23–38.
12. Kriesell HJ, Elwen SH, Nastasi A, Gridley T. Identification and characteristics of signature whistles in wild bottlenose dolphins (Tursiops truncatus) From Namibia. PLoS One. 2014;9(9).
13. Longden EG, Elwen SH, McGovern B, James BS, Embling CB, Gridley T. Mark-recapture of individually distinctive calls - A case study with signature whistles of bottlenose dolphins (Tursiops truncatus). J Mammal. 2020;101(5):1289–301.
14. Morisaka T, Shinohara M, Nakahara F, Akamatsu T, Morisaka T, Shinohara M, et al. Geographic variations in the whistles among three Indo-Pacific bottlenose dolphin. Fisheries Science. 2005;71:568–76.
15. Campbell G. Quantitative comparison of bottlenose dolphin (Tursiops truncatus) whistles from three geographic regions. 2004.
16. Hayes SA, Josephson E, Maze-Foley K, Rosel PE, Byrd B, Chavez-Rosales S, et al. US Atlantic and Gulf of Mexico Marine Mammal Stock Assessments [Internet]. NOAA Technical Memorandum. Woods Hole, MA; 2021. Available from: https://media.fisheries.noaa.gov/2021-07/Atlantic 2020 SARs Final.pdf?null%09
17. Garrod A, Fandel AD, Wingfield JE, Fouda L, Rice AN, Bailey H. Validating automated click detector dolphin detection rates and investigating factors affecting performance. J Acoust Soc Am. 2018 Aug;144(2):931–9.

18. Bailey H, Fandel AD, Silva KK, Gryzb E, McDonald E, Hoover AL, et al. Identifying and predicting occurrence and abundance of a vocal animal species based on individually specific calls. Ecosphere. 2021;12(8).
19. Gillespie D, White P, Caillat M, Gordon J. Development and Implementation of Automatic Classification of Odontocetes within PAMGUARD. 2011.
20. Janik VM, King SL, Sayigh LS, Wells RS. Identifying signature whistles from recordings of groups of unrestrained bottlenose dolphins (Tursiops truncatus). Mar Mamm Sci. 2013 Jan;29(January):109–22.
21. Gridley T, Cockcroft VG, Hawkins ER, Blewitt ML, Morisaka T, Janik VM. Signature whistles in free-ranging populations of Indo-Pacific bottlenose dolphins, Tursiops aduncus. Mar Mamm Sci. 2014;30(2):512–27.
22. Sportelli JJ, Jones BL, Ridgway SH. Non-linear phenomena: a common acoustic feature of bottlenose dolphin (Tursiops truncatus) signature whistles. Bioacoustics. 2022;
23. Deecke VB, Janik VM. Automated categorization of bioacoustic signals: Avoiding perceptual pitfalls. J Acoust Soc Am. 2006 Jan;119(1):645–53.
24. Barco S, Burt L, Deperte A, Digiovanni R. Prepared for the Maryland Department of Natural Resources Marine Mammal and Sea Turtle Sightings in the Vicinity of the Maryland Wind Energy Area All images-Riverhead Foundation for Marine Research and Preservation, Permit # 15575. 2013.
25. Bailey H, Lyubchich V, Wingfield JE, Fandel AD, Garrod A, Rice AN. Empirical evidence that large marine predator foraging behavior is consistent with area-restricted search theory. Ecology. 2019 Aug 1;100(8):1–9.
26. Oswald JN, Rankin S, Barlow JP, Lammers MO. A tool for real-time acoustic species identification of delphinid whistles. J Acoust Soc Am. 2007 Jul;122(1):587–95.
27. Fouda L, Wingfield JE, Fandel AD, Garrod A, Hodge KB, Rice AN, et al. Dolphins simplify their vocal calls in response to increased ambient noise. Biol Lett. 2018 Oct 1;14(10).
28. Venables WN, Ripley BD. Modern Applied Statistics with S. Fourth. New York, NY: Springer; 2002.
29. Kursa MB, Rudnicki WR. Feature selection with the boruta package. J Stat Softw. 2010;36(11):1–13.
30. Højsgaard S, Halekoh U. The R Package geepack for Generalized Estimating Equations. J Stat Softw. 2006;(2006):1–6.
31. Rodriguez LK, Fandel AD, Colbert BR, Testa JC, Bailey H. Spatial and temporal variation in the occurrence of bottlenose dolphins in the Chesapeake Bay, USA, using citizen science sighting data. PLoS One. 2021;16(5):e0251637.
32. Barco SG, Swingle WMark, McLellan WA, Harris RN, Pabst DAnn. Local abundance and distribution of bottlenose dolphins (Tursiops truncatus) in the nearshore water of Virginia Beach, Virginia. Mar Mamm Sci. 1999;15(2).
33. Hart PJ, Paxton K, Ibanez T, Tredinnick G, Sebastián-González E, Tanimoto-Johnson A. Acoustic niche partitioning in two tropical wet forest bird communities. bioRxiv. 2020;2020.08.17.253674.
34. Krause B, Farina A. Using ecoacoustic methods to survey the impacts of climate change on biodiversity. BIOC. 2016;195:245–54.

35. Luís AR, May-Collado LJ, Rako-Gospić N, Gridley T, Papale E, Azevedo A, et al. Vocal universals and geographic variations in the acoustic repertoire of the common bottlenose dolphin. Sci Rep. 2021;11(1):1–9.
36. Astrom G, Stolt B. Regional song dialects of the Ortolan Bunting Emberiza hortulana L. in Sweden. Ornis Svec. 1993;3:1–10.
37. Barker AJ, Veviurko G, Bennett NC, Hart DW, Mograby L, Lewin GR. Cultural transmission of vocal dialect in the naked mole-rat. Science (1979). 2021;371(6528):503–7.
38. Marler P, Tamura M. Song " Dialects " in Three Populations of White-Crowned Sparrows. Condor. 1962;64(5):368–77.
39. Daisy Kaplan J, Reiss D. Whistle acoustic parameters in atlantic spotted dolphins (Stenella frontalis) and bottlenose dolphins (Tursiops truncatus) in two locations in the bahamas and comparisons with other populations. Aquat Mamm. 2017;43(4):364–77.
40. Marley SA, Salgado Kent CP, Erbe C, Parnum IM. Effects of vessel traffic and underwater noise on the movement, behaviour and vocalisations of bottlenose dolphins in an urbanised estuary. Sci Rep. 2017 Dec 1;7(1).
41. van Ginkel C, Becker DM, Gowans S, Simard P. Whistling in a noisy ocean: Bottlenose dolphins adjust whistle frequencies in response to real-time ambient noise levels. Bioacoustics. 2018 Aug 24;27(4):391–405.
42. Picciulin M, Sebastianutto L, Codarin A, Calcagno G, Ferrero EA. Brown meagre vocalization rate increases during repetitive boat noise exposures : A possible case of vocal compensation. Journal of the Acoustical Society of America. 2012;132.
43. Hotchkin CF, Parks SE, Weiss DJ. Vocal modifications in primates: Effects of noise and behavioral context on vocalization structure. Proceedings of Meetings on Acoustics. 2013;19(010061).
44. Fournet MEH, Silvestri M, Clark CW, Klinck H, Rice AN. Limited vocal compensation for elevated ambient noise in bearded seals : implications for an industrializing Arctic Ocean. Proc Biol Sci. 2021;288.
45. Miksis-Olds JL, Tyack PL. Manatee (Trichechus manatus) vocalization usage in relation to environmental noise levels. Journal of the Acoustical Society of America. 2009;125:1806–15.
46. Krahforst CS, Sprague MW, Luczkovich JJ. The impact of vessel noise on oyster toadfish (Opsanus tau) communication. Proceedings of Meetings on Acoustics. 2016;27(010031).
47. Templeton CN, Zollinger SA, Brumm H. Traffic noise drowns out great tit alarm calls. Current Biology. 2016;26(22):R1173–4.
48. May-Collado LJ, Wartzok D. A Comparison of Bottlenose Dolphin Whistles in the Atlantic Ocean: Factors Promoting Whistle Variation. J Mammal. 2008;89(5):1229–40.
49. Ansmann IC, Goold JC, Evans PGH, Simmonds M, Keith SG. Variation in the whistle characteristics of short-beaked common dolphins, Delphinus delphis, at two locations around the British Isles. Journal of Marine Biological Assessment, UK. 2007;87:19–26.
50. Weilgart LS. The impacts of anthropogenic ocean noise on cetaceans and implications for management. Can J Zool. 2007;85(11):1091–116.

51. Marten K, Quine D, Marler P. Sound Transmission and Its Significance for Animal Vocalization: II. Tropical Forest Habitats [Internet]. Vol. 2, Sociobiology. 1977. Available from: https://about.jstor.org/terms

1. Janik VM, Sayigh LS. Communication in bottlenose dolphins: 50 years of signature whistle research. J Comp Physiol. 2013 Jun;199(6):479–89.

2. Filatova OA, Miller PJOO, Yurk H, Samarra FIPP, Hoyt E, Ford JKBB, et al. Killer whale call frequency is similar across the oceans, but varies across sympatric ecotypes. J Acoust Soc Am. 2015 Jul;138(1):251–7.

3. Ford JKB, Fisher HD. Killer whale (Orcinus orca) dialects as an indicator of stocks in British Columbia. 1982.

4. Scheer M, Hoffman B, Behr PI. Discrete pod-specific call repertoires among short-finned pilot whales (Globicephala macrorhynchus) off the SW coast of Tenerife, Canary Islands. In: World Marine Mammal Science Conference, 20-24 January, Monaco by European Cetacean Society and Society for Marine Mammalogy. Monaco; 1998. p. 1998.

5. Sayigh LS, Tyack PL, Wells RS, Scott MD. Signature whistles of free-ranging bottlenose dolphins Tursiops truncatus: stability and mother-offspring comparisons. Behav Ecol Sociobiol. 1990;26(4):247–60.

6. Caldwell MC, Caldwell DK. Individualized Whistle Contours in Bottle-nosed Dolphins (Tursiops truncatus). Nature. 1965;207(4995):434–5.

7. Cook MLH, Sayigh LS, Blum JE, Wells RS. Signature-whistle production in undisturbed free-ranging bottlenose dolphins (Tursiops truncatus). Proceedings of the Royal Society B: Biological Sciences. 2004 May 22;271(1543):1043–9.

8. Smolker RA, Mann J, Smuts BB. Use of signature whistles during separations and reunions by wild bottlenose dolphin mothers and infants. Behav Ecol Sociobiol. 1993;33(6):393–402.

9. Caldwell MC, Caldwell DK, Tyack PL. Review of the signature-whistle hypothesis for the Atlantic bottlenose dolphin. In: Leatherwood S, Reeves RR, editors. The bottlenose dolphin. New York, NY: Academic Press; 1990. p. 199–234.

10. Quick NJ, Janik VM. Bottlenose dolphins exchange signature whistles when meeting at sea. Proceedings of the Royal Society B: Biological Sciences. 2012;279(1738):2539–45.

11. Erbe C, Salgado-Kent C, de Winter S, Marley S, Ward R. Matching Signature Whistles with Photo-Identification of Indo-Pacific Bottlenose Dolphins (Tursiops aduncus) in the Fremantle Inner Harbour, Western Australia. Acoust Aust. 2020 Apr 1;48(1):23–38.

12. Kriesell HJ, Elwen SH, Nastasi A, Gridley T. Identification and characteristics of signature whistles in wild bottlenose dolphins (Tursiops truncatus) From Namibia. PLoS One. 2014;9(9).

13. Longden EG, Elwen SH, McGovern B, James BS, Embling CB, Gridley T. Mark-recapture of individually distinctive calls - A case study with signature whistles of bottlenose dolphins (Tursiops truncatus). J Mammal. 2020;101(5):1289–301.

14. Morisaka T, Shinohara M, Nakahara F, Akamatsu T, Morisaka T, Shinohara M, et al. Geographic variations in the whistles among three Indo-Pacific bottlenose dolphin. Fisheries Science. 2005;71:568–76.

15. Campbell G. Quantitative comparison of bottlenose dolphin (Tursiops truncatus) whistles from three geographic regions. 2004.

16. Hayes SA, Josephson E, Maze-Foley K, Rosel PE, Byrd B, Chavez-Rosales S, et al. US Atlantic and Gulf of Mexico Marine Mammal Stock Assessments [Internet]. NOAA

Technical Memorandum. Woods Hole, MA; 2021. Available from: https://media.fisheries.noaa.gov/2021-07/Atlantic 2020 SARs Final.pdf?null%09

17. Garrod A, Fandel AD, Wingfield JE, Fouda L, Rice AN, Bailey H. Validating automated click detector dolphin detection rates and investigating factors affecting performance. J Acoust Soc Am. 2018 Aug;144(2):931–9.
18. Bailey H, Fandel AD, Silva KK, Gryzb E, McDonald E, Hoover AL, et al. Identifying and predicting occurrence and abundance of a vocal animal species based on individually specific calls. Ecosphere. 2021;12(8).
19. Gillespie D, White P, Caillat M, Gordon J. Development and Implementation of Automatic Classification of Odontocetes within PAMGUARD. 2011.
20. Janik VM, King SL, Sayigh LS, Wells RS. Identifying signature whistles from recordings of groups of unrestrained bottlenose dolphins (Tursiops truncatus). Mar Mamm Sci. 2013 Jan;29(January):109–22.
21. Gridley T, Cockcroft VG, Hawkins ER, Blewitt ML, Morisaka T, Janik VM. Signature whistles in free-ranging populations of Indo-Pacific bottlenose dolphins, Tursiops aduncus. Mar Mamm Sci. 2014;30(2):512–27.
22. Sportelli JJ, Jones BL, Ridgway SH. Non-linear phenomena: a common acoustic feature of bottlenose dolphin (Tursiops truncatus) signature whistles. Bioacoustics. 2022;
23. Deecke VB, Janik VM. Automated categorization of bioacoustic signals: Avoiding perceptual pitfalls. J Acoust Soc Am. 2006 Jan;119(1):645–53.
24. Barco S, Burt L, Deperte A, Digiovanni R. Prepared for the Maryland Department of Natural Resources Marine Mammal and Sea Turtle Sightings in the Vicinity of the Maryland Wind Energy Area All images Riverhead Foundation for Marine Research and Preservation, Permit # 15575. 2013.
25. Bailey H, Lyubchich V, Wingfield JE, Fandel AD, Garrod A, Rice AN. Empirical evidence that large marine predator foraging behavior is consistent with area-restricted search theory. Ecology. 2019 Aug 1;100(8):1–9.
26. Oswald JN, Rankin S, Barlow JP, Lammers MO. A tool for real-time acoustic species identification of delphinid whistles. J Acoust Soc Am. 2007 Jul;122(1):587–95.
27. Fouda L, Wingfield JE, Fandel AD, Garrod A, Hodge KB, Rice AN, et al. Dolphins simplify their vocal calls in response to increased ambient noise. Biol Lett. 2018 Oct 1;14(10).
28. Venables WN, Ripley BD. Modern Applied Statistics with S. Fourth. New York, NY: Springer; 2002.
29. Kursa MB, Rudnicki WR. Feature selection with the boruta package. J Stat Softw. 2010;36(11):1–13.
30. Højsgaard S, Halekoh U. The R Package geepack for Generalized Estimating Equations. J Stat Softw. 2006;(2006):1–6.
31. Rodriguez LK, Fandel AD, Colbert BR, Testa JC, Bailey H. Spatial and temporal variation in the occurrence of bottlenose dolphins in the Chesapeake Bay, USA, using citizen science sighting data. PLoS One. 2021;16(5):e0251637.
32. Barco SG, Swingle WMark, McLellan WA, Harris RN, Pabst DAnn. Local abundance and distribution of bottlenose dolphins (Tursiops truncatus) in the nearshore water of Virginia Beach, Virginia. Mar Mamm Sci. 1999;15(2).

33. Hart PJ, Paxton K, Ibanez T, Tredinnick G, Sebastián-González E, Tanimoto-Johnson A. Acoustic niche partitioning in two tropical wet forest bird communities. bioRxiv. 2020;2020.08.17.253674.
34. Krause B, Farina A. Using ecoacoustic methods to survey the impacts of climate change on biodiversity. BIOC. 2016;195:245–54.
35. Luís AR, May-Collado LJ, Rako-Gospić N, Gridley T, Papale E, Azevedo A, et al. Vocal universals and geographic variations in the acoustic repertoire of the common bottlenose dolphin. Sci Rep. 2021;11(1):1–9.
36. Astrom G, Stolt B. Regional song dialects of the Ortolan Bunting Emberiza hortulana L. in Sweden. Ornis Svec. 1993;3:1–10.
37. Barker AJ, Veviurko G, Bennett NC, Hart DW, Mograby L, Lewin GR. Cultural transmission of vocal dialect in the naked mole-rat. Science (1979). 2021;371(6528):503–7.
38. Marler P, Tamura M. Song " Dialects " in Three Populations of White-Crowned Sparrows. Condor. 1962;64(5):368–77.
39. Daisy Kaplan J, Reiss D. Whistle acoustic parameters in atlantic spotted dolphins (Stenella frontalis) and bottlenose dolphins (Tursiops truncatus) in two locations in the bahamas and comparisons with other populations. Aquat Mamm. 2017;43(4):364–77.
40. Marley SA, Salgado Kent CP, Erbe C, Parnum IM. Effects of vessel traffic and underwater noise on the movement, behaviour and vocalisations of bottlenose dolphins in an urbanised estuary. Sci Rep. 2017 Dec 1;7(1).
41. van Ginkel C, Becker DM, Gowans S, Simard P. Whistling in a noisy ocean: Bottlenose dolphins adjust whistle frequencies in response to real-time ambient noise levels. Bioacoustics. 2018 Aug 24;27(4):391–405.
42. Picciulin M, Sebastianutto L, Codarin A, Calcagno G, Ferrero EA. Brown meagre vocalization rate increases during repetitive boat noise exposures : A possible case of vocal compensation. Journal of the Acoustical Society of America. 2012;132.
43. Hotchkin CF, Parks SE, Weiss DJ. Vocal modifications in primates: Effects of noise and behavioral context on vocalization structure. Proceedings of Meetings on Acoustics. 2013;19(010061).
44. Fournet MEH, Silvestri M, Clark CW, Klinck H, Rice AN. Limited vocal compensation for elevated ambient noise in bearded seals : implications for an industrializing Arctic Ocean. Proc Biol Sci. 2021;288.
45. Miksis-Olds JL, Tyack PL. Manatee (Trichechus manatus) vocalization usage in relation to environmental noise levels. Journal of the Acoustical Society of America. 2009;125:1806–15.
46. Krahforst CS, Sprague MW, Luczkovich JJ. The impact of vessel noise on oyster toadfish (Opsanus tau) communication. Proceedings of Meetings on Acoustics. 2016;27(010031).
47. Templeton CN, Zollinger SA, Brumm H. Traffic noise drowns out great tit alarm calls. Current Biology. 2016;26(22):R1173–4.
48. May-Collado LJ, Wartzok D. A Comparison of Bottlenose Dolphin Whistles in the Atlantic Ocean: Factors Promoting Whistle Variation. J Mammal. 2008;89(5):1229–40.
49. Ansmann IC, Goold JC, Evans PGH, Simmonds M, Keith SG. Variation in the whistle characteristics of short-beaked common dolphins, Delphinus delphis, at two locations around the British Isles. Journal of Marine Biological Assessment, UK. 2007;87:19–26.

50. Weilgart LS. The impacts of anthropogenic ocean noise on cetaceans and implications for management. Can J Zool. 2007;85(11):1091–116.

51. Marten K, Quine D, Marler P. Sound Transmission and Its Significance for Animal Vocalization: II. Tropical Forest Habitats [Internet]. Vol. 2, Sociobiology. 1977. Available from: https://about.jstor.org/terms

Reviewers' comments:

Reviewer's Responses to Questions
Comments to the Author

1. Is the manuscript technically sound, and do the data support the conclusions?

The manuscript must describe a technically sound piece of scientific research with data that supports the conclusions. Experiments must have been conducted rigorously, with appropriate controls, replication, and sample sizes. The conclusions must be drawn appropriately based on the data presented.
Reviewer #1: Partly
Reviewer #2: No

---

2. Has the statistical analysis been performed appropriately and rigorously?
Reviewer #1: Yes
Reviewer #2: Yes

---

3. Have the authors made all data underlying the findings in their manuscript fully available?

The PLOS Data policy requires authors to make all data underlying the findings described in their manuscript fully available without restriction, with rare exception (please refer to the Data Availability Statement in the manuscript PDF file). The data should be provided as part of the manuscript or its supporting information, or deposited to a public repository. For example, in addition to summary statistics, the data points behind means, medians and variance measures should be available. If there are restrictions on publicly sharing data—e.g. participant privacy or use of data from a third party—those must be specified.
Reviewer #1: Yes
Reviewer #2: No

---

4. Is the manuscript presented in an intelligible fashion and written in standard English?

PLOS ONE does not copyedit accepted manuscripts, so the language in submitted articles must be clear, correct, and unambiguous. Any typographical or grammatical errors should be corrected at revision, so please note any specific errors here.
Reviewer #1: Yes
Reviewer #2: Yes

---

5. Review Comments to the Author

Please use the space provided to explain your answers to the questions above. You may also include additional comments for the author, including concerns about dual publication, research ethics, or publication ethics. (Please upload your review as an attachment if it exceeds 20,000 characters)
Reviewer #1: Comments on Reviewed Manuscript.
Title: Vocal signatures affected by population identity and environmental sound levels

ID No: PONE-D-23-09444

Summary: The manuscript proposes methods to estimate the minimum numbers of the bottlenose dolphins (bd) in the Chesapeake Bay and waters of coastal Maryland and determine differences in the vocalizations of the bd from these sites (if they are site-specific) by comparing them with the vocalizations of bd from other regions. Also, the authors checked if individual bd change their signature in response to ambient sound conditions. Existing models on R are used for the analysis of recorded acoustics data.

Comments:

Kindly find below my comments/suggestions:

1. Line 33-33. This looks like an incomplete statement. I would rather suggest you state that passive acoustic monitoring (PAM) is a valuable technique for monitoring vocalizing species. Also, a sentence or 2 to define PAM may be helpful. This open-access review article on marine mammal vocalization may be helpful https://ieeexplore.ieee.org/abstract/document/9110497

- Thank you for the suggestion. A sentence on passive acoustic monitoring has been added. "Passive acoustic monitoring uses microphones (referred to as hydrophones in the underwater environment) to detect the sounds of vocalizing organisms." (line 35-36)

2. There are inconsistencies in the reference style adopted in the manuscript. Some seem APA (line 36 (Janik & Sayigh, 2013)), while others seem to be IEEE line 73 (14), Line 90 (16,17). Authors should stick to one (approved by the journal).

- Thank you for this correction. All citations are now Vancouver.

3. Introduction: a little more details should be given on PAM. The characteristics of dolphins' signatures and how they differ on an individual basis should be provided.

- I have inserted "Passive acoustic monitoring uses microphones (referred to as hydrophones in the underwater environment) to detect the sounds of vocalizing organisms." (line 35-36) and "These signature whistles comprise more than half of the whistles that wild bottlenose dolphins produce (7,8). They are formed in the first year of the dolphin's life (9) and remain relatively stable (5,9)." (line 42-44)

4. Figure 1: A clearer map of the study area should be provided. Also, in the explanation of the areas being investigated in this study, the authors should clearly state the two regions on the map. Also, did the author carry out new acoustic recordings for all regions or it uses already available data for some of the sites (as seems to be the explanation on Lines 72-94)? A distinction between sites/regions, as they relate to the manuscript, would make the manuscript clearer to readers.

- The resolution of the map has been increased and I have also underlined the sites from previous studies. I have also included additional details about recordings from previous studies to supplement Table 1. "Previous recordings at Site 1 occurred between June and August in 2017 and July to October in 2018. At Site 2, recordings occurred between July and September of 2016, January to April and June to October in 2017, and June to December in 2018. Site 3 recordings occurred between June and August in 2018." (line

82-85)

5. What does 14 mean in line 73?

- This was an incorrectly formatted reference. Thank you for the correction.

6. Line 86-87 states: Signature whistles were considered reoccurrences when they occurred on different days or in different locations.
This needs to be explained further.

- I have explained this further so the line reads "When the same signature whistle was identified at a different location or on a different day than the first instance, it was considered a reoccurrence of that individual signature whistle indicating the same dolphin had been detected again." (line 110-113)

7. Lines 121-130 state that: To understand whether bottlenose dolphins alter their signature whistles in response to……
How were the changes in ambient level determined/measured concerning the selected signature whistles? This needs to be buttressed further (particularly in the discussion of the results).

- To clarify this point, I have moved up the sentence at the end of this paragraph so it now reads "Ambient sound levels were calculated in MATLAB (MathWorks, Natick, Massachusetts, USA) as the relative broadband (up to 24 kHz, given the sampling rate of 48 kHz) sound pressure level (SPL; dB re 1µPa root-mean-square (rms)) during the recording in which the signature whistle occurred (two or five minutes in duration). Sound levels were also calculated for the duration of each acoustic deployment to determine overall ambient sound levels." (line 159-164) and in the results "When changes in signature whistle characteristics were modeled in relation to the ambient sound levels during which they occurred, only the duration of the signature whistles significantly varied in relation to changing ambient sound levels, decreasing as sound levels increased (Estimate = -0.005, S.E. = 0.003, Wald = 4.08, p = 0.04). All other signature whistle characteristics did not vary between reoccurrences when sound levels varied. (line 263-268)

8. Results section: Lines 134-137 need further clarification, with further details on the results in Table 1.

- We have added context to these lines to read: "A total of 1,172 unique signature whistles were detected at coastal Site 1, 327 at coastal Site 2, and 19 at Site 3 in the Bay (18). A total of 333 unique signature whistles were detected at Site 4 in the Potomac River in the Chesapeake Bay, and 37 at the offshore site, Site 5 (Table 1). An individuals' dolphin's presence is indicated by the presence of its signature whistle, so these counts of unique signature whistles represent a minimum abundance of bottlenose dolphins occurring at each site." (lines 174-179) and "For the subset of signature whistles for which whistle

characteristics were measured Table 1), the distribution of these characteristics (start, end, maximum, minimum, and delta frequencies, duration, number of local extrema) were non-parametric (Shapiro-Wilk test; $p < 0.05$).” (lines 186-189)

9. Figures 2-6: Clearer figures (particularly the labels & values) should be provided. The present Figures are not readable!

- Thank you. We have updated the figures.

10. Line 149: sqrt transformation, write in full at 1st mention.

- Thank you. We have spelled it out.

11. Line 152 7 others that follow. What are the units of the frequencies and other parameters?

- Thank you for this question. These numbers are the result of the MANOVA tests, not the values indicating the varied frequencies, therefore it is not appropriate to include units here, but we have referred to them in the Figures.

12. Lines 295-298: from ‘These findings…..to the end. The statement looks ambiguous and incomplete. Consider rephrasing with the clear meaning of the intended message. In motivating the need for effective management of the ocean due to increasing activities, more details should be provided by citing relevant related literature.

- We have rearranged this section to read: “Increasing sound levels in the ocean may continue to cause changes in dolphin calls leading to information loss (51) and reducing cohesion amongst dolphin groups, including mother-calf pairs, who use signature whistles to facilitate reunions (8). Sound levels in this region will rise due to development of offshore wind energy, coastal development, bridge construction, and increasing vessel traffic. It is particularly important to understand how individuals from particular populations may be exposed to sound from various human activities and hence how they may be impacted by masking of calls and potential disruption to their communication that could affect their fitness. The use of signature whistles to track individuals can also aid in determining how long individuals take to return to an area and resume normal behaviors after exposure to elevated sound levels. Such information will be vital for management of this region to determine the potential effects of, responses to, and recovery from anthropogenic sound pollution (50).” (lines 339-352)

Reviewer #2: I appreciate the dataset that was curated for this manuscript and know all of the hard work that goes into these sorts of large data analyses. Unfortunately, I do not recommend this manuscript for publication at this time. The main reasons are with the methodology: the methods are not the same for all sites, the methods are not described in enough detail for SIGID, Artwarp thresholds, how many of the same whistle type were allowed at each site, the random forrest classifiers used to classify bottlenose from common dolphins whistles, and the low

frequency sampling rate assessing things like maximum whistle frequency and broadband ambient noise levels. I think that with some care there can be some interesting findings from the robust dataset if the authors are able to consider these points.
Line Item Edits

Line 35: This is worded very strangely, consider reworking to just say that marine mammals are very vocal.

- Thank you. We have rephrased as suggested. “In the marine environment, one of the most vocal groups of organisms are marine mammals, which produce a variety of calls.” (lines 36-39)

Line 38: Caldwell and Caldwell 1965 founded signature whistles not Sayigh 1990

- We have corrected this, thank you.

Line 80. Why were all of these methods different? 8:2 and 9:1 duty cycle, visual observations compared to the whistle and moan detector?
Please include your own methods on identifying signature whistles from recordings using SIGID and Artwarp. These methods need to stand alone without needing to refer to other papers for operational definitions and thresholds used. For example what similarity threshold did you use to consider a signature whistle a reoccurrence.
A 48 kHz sampling rate gives a 24kHz Nyquist cut off. Dolphin whistles can range far above this range (up to over 40kHz). This needs to be clearly identified as a limitation of the study and may have resulted in distorted whistle feature data.

- These methods varied because the needs and recording conditions at different sites and time periods varied. The memory/battery capacity of the different recorders that were deployed in the shallower Bay waters compared to the deeper offshore site and the required deployment period between recovery cruises dictated the necessary duty cycle. We have clarified that some sites also had longer deployments, so recording needs varied.
    - “Due to longer deployments at Sites 4 and 5, , the Snap was duty-cycled for two minutes on and eight minutes off at Site 4 and one minute on and nine minutes off at Site 5..” (lines 92-94)

  We have also clarified that observers were only present at Site 4, so those criteria were only used for that site. At all sites, methods for narrowing down hours with possible signature whistles were used- whether focusing on the days with visual observations or use of the Whistle and Moan Detector.

  “At Site 4, a subsample of days were analyzed because of the high rate of dolphin presence at this location. At Site 5 (as with Sites 1 and 2), the PAMGUARD Whistle and Moan Detector (19) was utilized to determine hours with possible dolphin presence and these hours were then manually searched for signature whistles with high signal-to-noise ratios.” (lines 94-99)
- We thank the reviewer- we have included the frequency limitation as a caveat in the Discussion. “Additionally, because sounds with frequencies above 24 kHz could not be

detected in our recordings, higher frequency signature whistles would have been missed in our analyses [39].” (lines 318-320)

Line 84 you switch to using numbered citations instead of name and date?

- We have remedied this error.

Line 94: I am concerned about the species classification portion of this. You say you used ROCCA for species classification but it performed poorly (no data) and then say you used random forest models to confirm species identity but don’t provide any information on those models, their previous success rate or how you confirmed that those were successful here. When you identified a signature whistle, did you analyze multiple iterations of that whistle or just 1 example? What if one dolphin is hanging around one site for a long time whistling, does their one unqiue signature whistle end up contributing much of the whistle data?

- We understand this concern. We have included text below to elaborate on these results. “During the summer at the most inshore site (Site 1), bottlenose dolphins are the most likely species to be detected (25). In recordings from 21 hours on 16 days during the summer at Site 1, the ROCCA algorithm classified 61% of whistles as “Ambiguous” (n = 576), 13% as striped dolphin (*Stenella coeruleoalba*; n = 121*)*, 11% as bottlenose dolphins (n = 103), 8% as common dolphins (*Delphinus delphis*, n = 74), 4% as Clymene dolphin (*Stenella clymene*, n = 39*)*, 2 as Atlantic spotted dolphin (*Stenella frontalis*, n = 19*)*, and 1% as Pantropical spotted dolphin (*Stenella attenuate*, n.= 7). Clymene dolphins are typically found in waters deeper than 800 feet, and Pantropical spotted dolphins are not located in the Atlantic Ocean.” (lines 121-129)
- Additionally, we have added text to elaborate our methods so this paper stands alone. “Signature whistles were obtained using the SIGID criteria (1)-the same whistle repeated in a pattern of two or more whistles (7) within 1–10 s of one another and with a minimum length of 0.2 s (20,21). Whistle contours (shape of the whistle) were obtained using Beluga software (https://synergy.st-andrews.ac.uk/soundanalysis) within MATLAB (Math-Works, Natick, Massachusetts, USA). Whistles with low signal-to-noise ratio or abundant non-linear features (22) that obscured the shape of the whistle could not be included in the analysis. Beluga contours were analyzed within the adaptive resonance theory neural network (ART-warp; vigilance threshold of 96%; (23)) to identify when a whistle reoccurred. Identical signature whistles were not considered re-occurrences if they occurred on the same day at the same site. A human analyst verified all whistle re-occurrences.” (lines 99-108)

Line 125- What is a ‘clear’ signature whistle? Sportelli et al 2022 found that over 50% of signature whistles have non-linear features… Does this mean you did not include any whistles that had NLP?

- Whistles with non-linear features were included as long as analysts could determine the shape of the whistle. If too many non-linear features obscured the shape of the whistle, Beluga could not be used to obtain a contour, and thus the analysis could not be completed in ARTwarp. We have added text to indicate this. “Whistles with low signal-

to-noise ratio or abundant non-linear features [22] that obscured the shape of the whistle could not be included in the analysis." (lines 103-104)

Line 129- I am having a hard time with calling the ambient sound levels 'broad band' up to 24kHz when dolphin hearing goes into the 100s of kHz. Arguably there could have been any number of anthropogenic sound sources above the recording range.

- In this case, the term broadband was used to indicate that the sound level was calculated over the frequency range of the recorder, not to the hearing range of the species. We have rephrased this to read "as the broadband (up to 24 kHz, given the sampling rate of 48 kHz) sound pressure level" (line 159-161)

Discussion- what was the point of identifying signature whistles if the question was based on whistle features changing based on population, location and or ambient noise? A discussion of why not include all whistles would be helpful.

- Our research questions were: whether signature whistles varied by site indicating differential habitat use by individuals and potentially delineating populations; and whether animals adjusted their identity signature whistles in response to varied sound levels. Since this focuses on these unique, individually identifiable signature whistles, we did not include other types of whistle in our analysis.

If the signature whistle features were significantly different than a random selection of whistles at the same site, wouldn't this be an important result to suggest that the differences between sites may not be due to sites or ambient noise but just based on the fact that the different dolphins have different signature whistles?

- This is an excellent observation. However, because we did not compare a random sample of whistles from each site to signature whistles from those sites, we cannot make the conclusion that signature whistles vary from or are similar to other whistles from those locations or in those sound levels. We specifically compared signature whistles between sites and then examined whether there were any differences in repeated emissions of these signature whistles recorded during reoccurrences at other sites and under different ambient sound conditions.

Line 276: Your recording devices did not go up to the highest recorded whistle frequencies. See Jones et al., 2020 for a review or Kaplan and Reiss 2017 or Hiley et al. 2017 for evidence of whistles into the 40s of kHz.

- Thank you for these citations. We have added a citation to caveat our results. "Additionally, because sound with frequencies above 24 kHz could not be detected in our recordings, higher frequency signature whistles would have been missed in our analyses [35]." (lines 318-320)
