# Supplementary material for: Vocal signatures affected by population identity and environmental sound levels
Source: PLoS One. 2024 Apr 18;19(4):e0299250. doi: 10.1371/journal.pone.0299250 (PMC11025965; doi:10.1371/journal.pone.0299250)
Supplement: Attachment — Submitted filename: Review.docx [file pone.0299250.s001.docx]

I appreciate the dataset that was curated for this manuscript and know all of the hard work that goes into these sorts of large data analyses. Unfortunately, I do not recommend this manuscript for publication at this time. The main reasons are with the methodology: the methods are not the same for all sites, the methods are not described in enough detail for SIGID, Artwarp thresholds, how many of the same whistle type were allowed at each site, the random forrest classifiers used to classify bottlenose from common dolphins whistles, and the low frequency sampling rate assessing things like maximum whistle frequency and broadband ambient noise levels. I think that with some care there can be some interesting findings from the robust dataset if the authors are able to consider these points.

**Line Item Edits**

Line 35: This is worded very strangely, consider reworking to just say that marine mammals are very vocal.

Line 38: Caldwell and Caldwell 1965 founded signature whistles not Sayigh 1990

Line 80. Why were all of these methods different? 8:2 and 9:1 duty cycle, visual observations compared to the whistle and moan detector?

Please include your own methods on identifying signature whistles from recordings using SIGID and Artwarp. These methods need to stand alone without needing to refer to other papers for operational definitions and thresholds used. For example what similarity threshold did you use to consider a signature whistle a reoccurrence.

A 48 kHz sampling rate gives a 24kHz Nyquist cut off. Dolphin whistles can range far above this range (up to over 40kHz). This needs to be clearly identified as a limitation of the study and may have resulted in distorted whistle feature data.

Line 84 you switch to using numbered citations instead of name and date?

Line 94: I am concerned about the species classification portion of this. You say you used ROCCA for species classification but it performed poorly (no data) and then say you used random forest models to confirm species identity but don’t provide any information on those models, their previous success rate or how you confirmed that those were successful here.

When you identified a signature whistle, did you analyze multiple iterations of that whistle or just 1 example? What if one dolphin is hanging around one site for a long time whistling, does their one unqiue signature whistle end up contributing much of the whistle data?

Line 125- What is a ‘clear’ signature whistle? Sportelli et al 2022 found that over 50% of signature whistles have non-linear features… Does this mean you did not include any whistles that had NLP?

Line 129- I am having a hard time with calling the ambient sound levels ‘broad band’ up to 24kHz when dolphin hearing goes into the 100s of kHz. Arguably there could have been any number of anthropogenic sound sources above the recording range.

Line 159- The word site keeps getting pasted into the sentence

Discussion- what was the point of identifying signature whistles if the question was based on whistle features changing based on population, location and or ambient noise? A discussion of why not include all whistles would be helpful.

If the signature whistle features were significantly different than a random selection of whistles at the same site, wouldn't this be an important result to suggest that the differences between sites may not be due to sites or ambient noise but just based on the fact that the different dolphins have different signature whistles?

Line 276: Your recording devices did not go up to the highest recorded whistle frequencies. See Jones et al., 2020 for a review or Kaplan and Reiss 2017 or Hiley et al. 2017 for evidence of whistles into the 40s of kHz.
