# Supplementary material for: Vocal signatures affected by population identity and environmental sound levels
Source: PLoS One. 2024 Apr 18;19(4):e0299250. doi: 10.1371/journal.pone.0299250 (PMC11025965; doi:10.1371/journal.pone.0299250)
Supplement: Attachment — Submitted filename: PLOS_ReviewResponseLetter_SWChars_Final.docx [file pone.0299250.s002.docx]

Reviewers' comments:

Reviewer's Responses to Questions

**Comments to the Author**

1. Is the manuscript technically sound, and do the data support the conclusions?

The manuscript must describe a technically sound piece of scientific research with data that supports the conclusions. Experiments must have been conducted rigorously, with appropriate controls, replication, and sample sizes. The conclusions must be drawn appropriately based on the data presented.

Reviewer #1: Partly

Reviewer #2: No

2. Has the statistical analysis been performed appropriately and rigorously?

Reviewer #1: Yes

Reviewer #2: Yes

3. Have the authors made all data underlying the findings in their manuscript fully available?

The [PLOS Data policy](http://www.plosone.org/static/policies.action#sharing) requires authors to make all data underlying the findings described in their manuscript fully available without restriction, with rare exception (please refer to the Data Availability Statement in the manuscript PDF file). The data should be provided as part of the manuscript or its supporting information, or deposited to a public repository. For example, in addition to summary statistics, the data points behind means, medians and variance measures should be available. If there are restrictions on publicly sharing data—e.g. participant privacy or use of data from a third party—those must be specified.

Reviewer #1: Yes

Reviewer #2: No

4. Is the manuscript presented in an intelligible fashion and written in standard English?

PLOS ONE does not copyedit accepted manuscripts, so the language in submitted articles must be clear, correct, and unambiguous. Any typographical or grammatical errors should be corrected at revision, so please note any specific errors here.

Reviewer #1: Yes

Reviewer #2: Yes

5. Review Comments to the Author

Please use the space provided to explain your answers to the questions above. You may also include additional comments for the author, including concerns about dual publication, research ethics, or publication ethics. (Please upload your review as an attachment if it exceeds 20,000 characters)

Reviewer #1: Comments on Reviewed Manuscript.
Title: Vocal signatures affected by population identity and environmental sound levels
ID No: PONE-D-23-09444
Summary: The manuscript proposes methods to estimate the minimum numbers of the bottlenose dolphins (bd) in the Chesapeake Bay and waters of coastal Maryland and determine differences in the vocalizations of the bd from these sites (if they are site-specific) by comparing them with the vocalizations of bd from other regions. Also, the authors checked if individual bd change their signature in response to ambient sound conditions. Existing models on R are used for the analysis of recorded acoustics data.

Comments:
Kindly find below my comments/suggestions:
1. Line 33-33. This looks like an incomplete statement. I would rather suggest you state that passive acoustic monitoring (PAM) is a valuable technique for monitoring vocalizing species. Also, a sentence or 2 to define PAM may be helpful. This open-access review article on marine mammal vocalization may be helpful <https://ieeexplore.ieee.org/abstract/document/9110497>

- Thank you for the suggestion. A sentence on passive acoustic monitoring has been added.

“Passive acoustic monitoring uses microphones (referred to as hydrophones in the underwater environment) to detect the sounds of vocalizing organisms.” (line 35-36)

2. There are inconsistencies in the reference style adopted in the manuscript. Some seem APA (line 36 (Janik & Sayigh, 2013)), while others seem to be IEEE line 73 (14), Line 90 (16,17). Authors should stick to one (approved by the journal).

- Thank you for this correction. All citations are now Vancouver.

3. Introduction: a little more details should be given on PAM. The characteristics of dolphins' signatures and how they differ on an individual basis should be provided.

- I have inserted “Passive acoustic monitoring uses microphones (referred to as hydrophones in the underwater environment) to detect the sounds of vocalizing organisms.” (line 35-36) and “These signature whistles comprise more than half of the whistles that wild bottlenose dolphins produce (7,8). They are formed in the first year of the dolphin’s life (9) and remain relatively stable (5,9).” (line 42-44)

4. Figure 1: A clearer map of the study area should be provided. Also, in the explanation of the areas being investigated in this study, the authors should clearly state the two regions on the map. Also, did the author carry out new acoustic recordings for all regions or it uses already available data for some of the sites (as seems to be the explanation on Lines 72-94)? A distinction between sites/regions, as they relate to the manuscript, would make the manuscript clearer to readers.

- The resolution of the map has been increased and I have also underlined the sites from previous studies. I have also included additional details about recordings from previous studies to supplement Table 1. “Previous recordings at Site 1 occurred between June and August in 2017 and July to October in 2018. At Site 2, recordings occurred between July and September of 2016, January to April and June to October in 2017, and June to December in 2018. Site 3 recordings occurred between June and August in 2018.” (line 82-85)

5. What does 14 mean in line 73?

- This was an incorrectly formatted reference. Thank you for the correction.

6. Line 86-87 states: Signature whistles were considered reoccurrences when they occurred on different days or in different locations.
This needs to be explained further.

- I have explained this further so the line reads “When the same signature whistle was identified at a different location or on a different day than the first instance, it was considered a reoccurrence of that individual signature whistle indicating the same dolphin had been detected again.” (line 110-113)

7. Lines 121-130 state that: To understand whether bottlenose dolphins alter their signature whistles in response to……
How were the changes in ambient level determined/measured concerning the selected signature whistles? This needs to be buttressed further (particularly in the discussion of the results).

- To clarify this point, I have moved up the sentence at the end of this paragraph so it now reads “Ambient sound levels were calculated in MATLAB (MathWorks, Natick, Massachusetts, USA) as the relative broadband (up to 24 kHz, given the sampling rate of 48 kHz) sound pressure level (SPL; dB re 1µPa root-mean-square (rms)) during the recording in which the signature whistle occurred (two or five minutes in duration). Sound levels were also calculated for the duration of each acoustic deployment to determine overall ambient sound levels.” (line 159-164) and in the results “When changes in signature whistle characteristics were modeled in relation to the ambient sound levels during which they occurred, only the duration of the signature whistles significantly varied in relation to changing ambient sound levels, decreasing as sound levels increased (Estimate = -0.005, S.E. = 0.003, Wald = 4.08, p = 0.04). All other signature whistle characteristics did not vary between reoccurrences when sound levels varied. (line 263-268)

8. Results section: Lines 134-137 need further clarification, with further details on the results in Table 1.

- We have added context to these lines to read: “A total of 1,172 unique signature whistles were detected at coastal Site 1, 327 at coastal Site 2, and 19 at Site 3 in the Bay (18). A total of 333 unique signature whistles were detected at Site 4 in the Potomac River in the Chesapeake Bay, and 37 at the offshore site, Site 5 (Table 1). An individuals’ dolphin’s presence is indicated by the presence of its signature whistle, so these counts of unique signature whistles represent a minimum abundance of bottlenose dolphins occurring at each site.” (lines 174-179) and “For the subset of signature whistles for which whistle characteristics were measured Table 1), the distribution of these characteristics (start, end, maximum, minimum, and delta frequencies, duration, number of local extrema) were non-parametric (Shapiro-Wilk test; p < 0.05).” (lines 186-189)

9. Figures 2-6: Clearer figures (particularly the labels & values) should be provided. The present Figures are not readable!

- Thank you. We have updated the figures.

10. Line 149: sqrt transformation, write in full at 1st mention.

- Thank you. We have spelled it out.

11. Line 152 7 others that follow. What are the units of the frequencies and other parameters?

- Thank you for this question. These numbers are the result of the MANOVA tests, not the values indicating the varied frequencies, therefore it is not appropriate to include units here, but we have referred to them in the Figures.

12. Lines 295-298: from ‘These findings…..to the end. The statement looks ambiguous and incomplete. Consider rephrasing with the clear meaning of the intended message. In motivating the need for effective management of the ocean due to increasing activities, more details should be provided by citing relevant related literature.

- We have rearranged this section to read: “Increasing sound levels in the ocean may continue to cause changes in dolphin calls leading to information loss (51) and reducing cohesion amongst dolphin groups, including mother-calf pairs, who use signature whistles to facilitate reunions (8). Sound levels in this region will rise due to development of offshore wind energy, coastal development, bridge construction, and increasing vessel traffic. It is particularly important to understand how individuals from particular populations may be exposed to sound from various human activities and hence how they may be impacted by masking of calls and potential disruption to their communication that could affect their fitness. The use of signature whistles to track individuals can also aid in determining how long individuals take to return to an area and resume normal behaviors after exposure to elevated sound levels. Such information will be vital for management of this region to determine the potential effects of, responses to, and recovery from anthropogenic sound pollution (50).” (lines 339-352)

**Reviewer #2**: I appreciate the dataset that was curated for this manuscript and know all of the hard work that goes into these sorts of large data analyses. Unfortunately, I do not recommend this manuscript for publication at this time. The main reasons are with the methodology: the methods are not the same for all sites, the methods are not described in enough detail for SIGID, Artwarp thresholds, how many of the same whistle type were allowed at each site, the random forrest classifiers used to classify bottlenose from common dolphins whistles, and the low frequency sampling rate assessing things like maximum whistle frequency and broadband ambient noise levels. I think that with some care there can be some interesting findings from the robust dataset if the authors are able to consider these points.
Line Item Edits

Line 35: This is worded very strangely, consider reworking to just say that marine mammals are very vocal.

- Thank you. We have rephrased as suggested. “In the marine environment, one of the most vocal groups of organisms are marine mammals, which produce a variety of calls.” (lines 36-39)

Line 38: Caldwell and Caldwell 1965 founded signature whistles not Sayigh 1990

- We have corrected this, thank you.

Line 80. Why were all of these methods different? 8:2 and 9:1 duty cycle, visual observations compared to the whistle and moan detector?
Please include your own methods on identifying signature whistles from recordings using SIGID and Artwarp. These methods need to stand alone without needing to refer to other papers for operational definitions and thresholds used. For example what similarity threshold did you use to consider a signature whistle a reoccurrence.
A 48 kHz sampling rate gives a 24kHz Nyquist cut off. Dolphin whistles can range far above this range (up to over 40kHz). This needs to be clearly identified as a limitation of the study and may have resulted in distorted whistle feature data.

- These methods varied because the needs and recording conditions at different sites and time periods varied. The memory/battery capacity of the different recorders that were deployed in the shallower Bay waters compared to the deeper offshore site and the required deployment period between recovery cruises dictated the necessary duty cycle. We have clarified that some sites also had longer deployments, so recording needs varied.
  - “Due to longer deployments at Sites 4 and 5, , the Snap was duty-cycled for two minutes on and eight minutes off at Site 4 and one minute on and nine minutes off at Site 5..” (lines 92-94)

We have also clarified that observers were only present at Site 4, so those criteria were only used for that site. At all sites, methods for narrowing down hours with possible signature whistles were used- whether focusing on the days with visual observations or use of the Whistle and Moan Detector.

“At Site 4, a subsample of days were analyzed because of the high rate of dolphin presence at this location. At Site 5 (as with Sites 1 and 2), the PAMGUARD Whistle and Moan Detector (19) was utilized to determine hours with possible dolphin presence and these hours were then manually searched for signature whistles with high signal-to-noise ratios.” (lines 94-99)

- We thank the reviewer- we have included the frequency limitation as a caveat in the Discussion. “Additionally, because sounds with frequencies above 24 kHz could not be detected in our recordings, higher frequency signature whistles would have been missed in our analyses [39].” (lines 318-320)

Line 84 you switch to using numbered citations instead of name and date?

- We have remedied this error.

Line 94: I am concerned about the species classification portion of this. You say you used ROCCA for species classification but it performed poorly (no data) and then say you used random forest models to confirm species identity but don’t provide any information on those models, their previous success rate or how you confirmed that those were successful here.
When you identified a signature whistle, did you analyze multiple iterations of that whistle or just 1 example? What if one dolphin is hanging around one site for a long time whistling, does their one unqiue signature whistle end up contributing much of the whistle data?

- We understand this concern. We have included text below to elaborate on these results.

“During the summer at the most inshore site (Site 1), bottlenose dolphins are the most likely species to be detected (25). In recordings from 21 hours on 16 days during the summer at Site 1, the ROCCA algorithm classified 61% of whistles as “Ambiguous” (n = 576), 13% as striped dolphin (*Stenella coeruleoalba*; n = 121*)*, 11% as bottlenose dolphins (n = 103), 8% as common dolphins (*Delphinus delphis*, n = 74), 4% as Clymene dolphin (*Stenella clymene*, n = 39*)*, 2 as Atlantic spotted dolphin (*Stenella frontalis*, n = 19*)*, and 1% as Pantropical spotted dolphin (*Stenella attenuate*, n.= 7). Clymene dolphins are typically found in waters deeper than 800 feet, and Pantropical spotted dolphins are not located in the Atlantic Ocean.” (lines 121-129)

- Additionally, we have added text to elaborate our methods so this paper stands alone. “Signature whistles were obtained using the SIGID criteria (1)-the same whistle repeated in a pattern of two or more whistles (7) within 1–10 s of one another and with a minimum length of 0.2 s (20,21). Whistle contours (shape of the whistle) were obtained using Beluga software (<https://synergy.st-andrews.ac.uk/soundanalysis>) within MATLAB (Math-Works, Natick, Massachusetts, USA). Whistles with low signal-to-noise ratio or abundant non-linear features (22) that obscured the shape of the whistle could not be included in the analysis. Beluga contours were analyzed within the adaptive resonance theory neural network (ART-warp; vigilance threshold of 96%; (23)) to identify when a whistle reoccurred. Identical signature whistles were not considered re-occurrences if they occurred on the same day at the same site. A human analyst verified all whistle re-occurrences.” (lines 99-108)

Line 125- What is a ‘clear’ signature whistle? Sportelli et al 2022 found that over 50% of signature whistles have non-linear features… Does this mean you did not include any whistles that had NLP?

- Whistles with non-linear features were included as long as analysts could determine the shape of the whistle. If too many non-linear features obscured the shape of the whistle, Beluga could not be used to obtain a contour, and thus the analysis could not be completed in ARTwarp. We have added text to indicate this. “Whistles with low signal-to-noise ratio or abundant non-linear features [22] that obscured the shape of the whistle could not be included in the analysis.” (lines 103-104)

Line 129- I am having a hard time with calling the ambient sound levels ‘broad band’ up to 24kHz when dolphin hearing goes into the 100s of kHz. Arguably there could have been any number of anthropogenic sound sources above the recording range.

- In this case, the term broadband was used to indicate that the sound level was calculated over the frequency range of the recorder, not to the hearing range of the species. We have rephrased this to read “as the broadband (up to 24 kHz, given the sampling rate of 48 kHz) sound pressure level” (line 159-161)

Discussion- what was the point of identifying signature whistles if the question was based on whistle features changing based on population, location and or ambient noise? A discussion of why not include all whistles would be helpful.

- Our research questions were: whether signature whistles varied by site indicating differential habitat use by individuals and potentially delineating populations; and whether animals adjusted their identity signature whistles in response to varied sound levels. Since this focuses on these unique, individually identifiable signature whistles, we did not include other types of whistle in our analysis.

If the signature whistle features were significantly different than a random selection of whistles at the same site, wouldn't this be an important result to suggest that the differences between sites may not be due to sites or ambient noise but just based on the fact that the different dolphins have different signature whistles?

- This is an excellent observation. However, because we did not compare a random sample of whistles from each site to signature whistles from those sites, we cannot make the conclusion that signature whistles vary from or are similar to other whistles from those locations or in those sound levels. We specifically compared signature whistles between sites and then examined whether there were any differences in repeated emissions of these signature whistles recorded during reoccurrences at other sites and under different ambient sound conditions.

Line 276: Your recording devices did not go up to the highest recorded whistle frequencies. See Jones et al., 2020 for a review or Kaplan and Reiss 2017 or Hiley et al. 2017 for evidence of whistles into the 40s of kHz.

- Thank you for these citations. We have added a citation to caveat our results. “Additionally, because sound with frequencies above 24 kHz could not be detected in our recordings, higher frequency signature whistles would have been missed in our analyses [35].” (lines 318-320)
