# Supplementary material for: Vocal signatures affected by population identity and environmental sound levels
Source: PLoS One. 2024 Apr 18;19(4):e0299250. doi: 10.1371/journal.pone.0299250 (PMC11025965; doi:10.1371/journal.pone.0299250)
Supplement: Attachment — Submitted filename: Reviewers comment_PONE-D-23-09444R1.docx [file pone.0299250.s003.docx]

**Comments on Reviewed Manuscript (Revision 1).**

**Title:** Vocal signatures affected by population identity and environmental sound levels

**ID No:** PONE-D-23-09444R1

**Comments:**

The authors have made considerable efforts to improve the quality of the previous manuscript. However, there are still some areas that need to be checked/addressed. Kindly find below my comments/suggestions:

1. I have submitted an annotated version of the revised manuscript where I included some comments and suggestions.
2. While a consistent referencing style has been adopted, the formatting of the referencing is still not up to standard. I don’t know maybe this is as a result of converting the original template to PDF. The authors should check to be sure all is okay. For instance, check line 105 where you have a full-stop sign before the reference: A human analyst verified all whistle re-occurrences. (1,20).

Also there should be spacing between the ref nos.
