# Supplementary material for: Vocal signatures affected by population identity and environmental sound levels
Source: PLoS One. 2024 Apr 18;19(4):e0299250. doi: 10.1371/journal.pone.0299250 (PMC11025965; doi:10.1371/journal.pone.0299250)
Supplement: Attachment — Submitted filename: Response to reviewers comments_PONE-D-23-09444R1_23.12.18.docx [file pone.0299250.s005.docx]

Vocal signatures affected by population identity and environmental sound levels

**ID No:** PONE-D-23-09444R1

We thank the reviewers and editors for their time and thoughtful reviews of this manuscript. We have included our responses to their comments below. Please let us know if any additional edits are suggested. Thank you again.

Reviewer's Responses to Questions

**Comments to the Author**

**Reviewer #1:**

The authors have made considerable efforts to improve the quality of the previous manuscript. However, there are still some areas that need to be checked/addressed. I have attached 2 documents for the authors' attention.

Line 34: PAM is especially valuable for monitoring vocalizing species.

We have rephrased as suggested.

Line 35: This is not a correct definition of PAM.

PAM is for data recording/gathering, not for detection.

The readers will benefit/be well carried along with good opening to your paper.

see https://ieeexplore.ieee.org/abstract/document/9110497 or https://doi.org/10.1016/j.ecoinf.2022.101766 for reference.

We have corrected our definiteion and re-structured our introduction to flow from group to

individual acoustic indentification. We have also added a few lines as a more compelling

introduction.

Line 47: this sentence is unclear/incomplete. Kindly look into it.

We have rephrased to read “Distinct populations of bottlenose dolphins vary the

characterstics of their whistles (14,15), but variations in signature whistles have not yet

been investigated.” (line 54-57)

Line 54: Make your manuscript reading flow.....This study aims to 1) to determine ..aims to 2) whether? it should be 2)know/determine whether there are..... The same to the 3rd aim.

We have rephrased to read “This study aims to determine 1)…”

Line 66: Does this mean anyone can just put autonomous recorders in the study area?

For the majority of the area, that is true. We have clarified “No permits were required for

this work as there was no handling or interaction with the study species, but permission

was obtained from the leaseholders of the wind energy area for the deployment of

bottom-anchored autonomous recorders.” (line 77-80)

Line 105: check you reference format and other formating style.

Thank you. Corrected.

Line 114: is this a reference?

This is a reference to personal communications. The style has been corrected.

**Reviewer #3:**

Introduction
Line 32-33: could perhaps use another adjective to ‘challenging’ twice

Apologies, I do not see the duplicated word here.

34-35: These sentences could be edited/combined to help flow

We have combined them as suggested.

37: detection of individuals and population groups

Changed as requested

42: In killer whales (Orcinus orca)

Changed as requested

Line 36-45: perhaps consider rearranging so that all the information on calls unique to population groups, and then calls unique to individuals are together

Revised as requested. Thank you.

Line 50-52: either move the ‘1)’ to after ‘determine’ or add a verb after the ‘2)’ and ‘3)’

Moved determine to before the 1) as suggested.

Perhaps a sentence or two could be added about previous work using signature whistles previously, and if they have been used before in this way

We have added a sentence outlining previous studies in this region. “Previous studies in

this region investigated the presence and temporal patterns of bottlenose dolphins (17–20) and documented their signature whistles (21). This study aims to build upon that work and determine…” (line 61-63)

Methods
Line 66: What does the Garrod et al reference refer to – make this clear

We have removed this reference.

Line 73, 84: ‘described in 14’?, ‘methodology in 14’

We have revised line 73, and removed the reference from line 84.

Line 72- 77: these details may be clearer in a table, and is somewhat repetitive to lines 63-66
We have added a table as suggested (Table 2).

Line 73: What type of recorders were used at sites 1-3? Even if referencing another study, the basic details should be available to the reader here

Details on recorders have been included in Table 2.

Line 79: how was the duty cycle timing selected – was it purely to extend the recording to 2 months
Please be more specific with the timing of the recordings
We have specified that duty cycling was selected simply to prolong battery life.

Deployments were 3-6 months.

Line 82: Was any validation of the use of the PAMGUARD W&M detector performed to check accuracy. Were any times when calls were not indicated checked for presence to ensure accuracy and reliability of the detectors? This could be a major limitation in the call identification phase
After the PAMGUARD W&M detector indicated dolphin presence, he process of

selecting the signature whistles was entirely manula, so we are positive that these are true

detections. We have clarified this detail.

“Signature whistles were manually identified using the SIGID criteria” (line 115-117)

Line 88-90: were the sites and recording timing chosen because of this or was it coincidental?
Yes, we had prior knowledge that dolphins were present in this region at these times.

Line 91-94: same comment for PAMGUARD applies here for ROCCA – was it tested and verified in any way with this population, given known limitations and poor performance. You mention in lines 94-95 that there is poor performance in this analysis – so the reader needs details about how this was established

ROCCA was only used to determine whether it was a viable option for determining

which species were present in the recordings. However, it was found to be extremely

unreliable and not used in the final analyses. Including this analysis was recommended by

another reviewer.

Lines 96-97: Much more details on this process is needed. Is there any researcher validation in this process?
As mentioned above, including this analysis was only included to document that using

ROCCA was not a viable option in determining which species was present.

Line 101: perhaps good to state what you mean by ‘full state was clear’ means to you
We have removed this to improve clarity.

Line 101-104: How were these manual measurements taken?
We have clarified that these measurements were taken in Raven. Markers were manually

placed on the whistle.

Line 107: why push into parameteric processes?
We wanted to use a MANOVA to test whether the characteristics of whistles varied by

site, which are parametric analyses.

Line 112-114: would the random forest not be a better method here throughout and not just to establish input importance? More details of this process are needed, including more details about the input variables and their resolution
ROCCA is an algorithm that can be used in PAMGUARD. We have added additional

details on our analyses and rearranged for clarity. “To confirm that the species detected

were bottlenose, not common dolphin (*Delphinus delphis*) ), we tested the utility of

the Real-time Odontocete Call Classification Algorithm (ROCCA) (28) in

PAMGUARD.” (line 131-134)

Line 126: How did you define ‘changes in ambient sound levels’ – they are constantly changing
The sound level during the recording (2-5 minutes) was included in the GEE model and

modeled against the signature whistle characteristics. We have rephrased: “Generalized

estimating equations-generalized linear models (GEEs) in R (geepack package in R; (33)

were used to determine whether and which changes in signature whistles characteristics

were correlated with changes in ambient sound levels.” (line 193-196)

Line 128-129: How were ambient sound levels calculated?
“Ambient sound levels were calculated in MATLAB (MathWorks, Natick,

Massachusetts, USA) as the relative broadband (up to 24 kHz, given the sampling rate of

48 kHz) sound pressure level (SPL; dB re 1µPa root-mean-square (rms)) during the

recording in which the signature whistle occurred (two or five minutes in duration).” (line

188-191)

Results
Line 147-148: what does ‘become normally distributed’ mean?
We have rephrased to read “were normally distributed”. (line 218) We meant to indicate

whether the characteristics were normally distributed after transformation.

Line 154: what does number of extrema mean, and what are you using it to indicate?
The number of extrema is a count of the local minima and maxima and can be considered

a proxy for complexity. We have clarified “A MANOVA indicated that start (F_4,352_ =

2.71, p = 0.03), end (F_4,352_ = 4.52, p = 0.01), and minimum frequencies (F_4,352_ = 10.3, p <

0.01) as well as duration (F_4,352_ = 9.36, p < 0.01) and number of extrema (F_4,352_ = 7.28, p

< 0.01), or complexity, of signature whistles varied significantly by site.” (line 224-227)

Some formatting/text issues on Line 159
I believe this is a result of Word’s formatting. It should be fixed when formatted by the

Journal.

Line 170-175: if there are significant differences by site then how would this impact your conclusions based on ambient sound levels etc.
- Are dolphin groups known to be site loyal – what does this result say to your group/individual tracking question
Dolphins are generally not loyal to specific sites in this region, though offshore

bottlenose dolphins do not generally come to more inshore sites.

Line 185-187: What do you mean here?
We have struck this portion, as we believe that we indicated our methods in the sentences

before in which we state the transformations of the variables.

Figure 5: more details in the methods section would help with the interpretation of these results and knowing what was an input variable and what was a shadow variable – and how that was selected and included into the model
I have added details in the methods to read: “The Boruta package in R (32) randomizes

the data for each factor (called shadow data) then utilizes random forest models with the

data and shadow data to assess factor importance in determining an identity (e.g. site or

region).” (line 178-181)

How would a variable decrease the accuracy of the model – why would it not just be a none significant/none impactful factor?
We have rephrased to “When the start frequency and number of extrema were included in

the model, the model was less accurate when determining the region to which a signature

whistle belonged (Fig 5b).” (line 257-260)

Although Figure 6 is informative – I wonder whether the details in the figure and the text (Lines 207-211) would be better displayed in a table or matrix of some kind
We have used this plot to illustrate the variation in sound levels at each site and whether

the sounds levels varied significantly between each. We would prefer to keep this plot.

Line 218-221: More details are needed here

We have added “Reoccurences of whistles indicate that dolphins were detected at

different sites on different days.” (line 279-280) to clarify the significance of

reoccurences.

Discussion
Line 225: This may be true, except you have identified that these unique whistles at different sites – with the calls at the deeper locations also heard at the very coastal locations
We have added additional details to the first sentence. “We determined that bottlenose

dolphins reoccurred through time and space and that the characteristics of bottlenose

dolphin signature whistles significantly varied by site and region.” (310-311)

Line 226-228: This should be a separate discussion, as this may or may not be site related
We have expanded upon this below, but wanted to provide an overview of the discussion.

Line 229-230: I am not sure how you have demonstrated this with your results as the paper/results are presented currently. Perhaps the site differences and cross over between sites (or not) should be stated more strongly/clearer – and the loyalty of population groups to sites be highlighted from the outset if this is indeed the case (perhaps not given comments in Lines 244-245)
We have expanded upon the reocurrences of the whistles between sites.

“In total, 201 unique signature whistles reoccurred a total of 252 times at the five

different sites (Fig. 7). Reoccurences of whistles indicate that dolphins were detected at

different sites on different days. Signature whistles most often reoccurred at the inshore sites, Sites 1 and 2 (n = 49; 15% of all unique signature whistles from Site 2) and between the most inshore site, Site 1, and the lower Bay site, Site 4 (n = 30, 9% of unique signature whistles from Site 4; Fig. 3.9).

Of the 19 unique signature whistles detected at the upper Bay site (Site 3), 26% (n = 5) were also detected at the lower Bay site (Site 4;Fig. 7). At the lower Bay site, 24 of the total 333 unique signature whistles identified reoccurred 27 times within the site on different days indicating that dolphins may be spending several days within the Bay and were detected as they enter and exit the Bay.

Of the 37 unique signature whistles detected at Site 5, only three signature whistles reoccurred within the site. Signature whistles from the offshore Site 5 were also detected three times at Site 2 (two of which were the same whistle; 5% of all unique signature whistles from Site 5) and one was detected at Site 1 (3%; Fig. 7). “ (line 278-292)

Line 256: why were these sites and photo-ID not used in this study?

Photo-ID was not available for this study, and acoustic recordings were not available

when the study with photo-ID were conducted.

Line 170: why was 120 dB used as your threshold for ‘high ambient sound levels’ – perhaps give a reference

We have added a reference to the National Research Council’s 2005 paper on

biologically significant effects of noise.

Line 273: so whistles may increase with volume when other/more conspecific calls are present? This is a very different conclusion to purely stating ‘increased ambient sound levels’ increased
We did not assess the volume of the calls, only the ambient sound level in the two or five

minute recording in which the signature whistle occurred. Thus, ambient sound levels in

these recordings could be due to other whistles during the recording. We have added text

to read “Because our calculation of ambient sound levels did not exclude other whistles,

higher sound levels could also be indicative of more whistles within the 2 or 5-minute

recordings.” (line 358-360)

Line 285: what did you classify as low frequency sounds?

These studies reference fish calls and sounds below the frequencies of bottlenose dolphin

whistles, so we have specified in the text. “Whistles with higher frequencies (27,41) and

fewer extrema (27,40) are common where low frequency (generally below 3 kHz)

ambient sound levels are higher.” (line 370-372)

**Reviewer #4:** 
L75 - you should also mention the reference number (18)
Added. Thanks!

L103 - reference 23 should be inside brackets "... 96%; (23)) to ...."
Should be corrected now.

L115- This needs to be further explained. You say your results are poor in what concerns spcs Identification and then just say you relied your certainty of the species using random forest (line 125-127) from a different study on the same populations. Can you explain a bit further on those results? I should not have to go to the reference to look for your study results.
We have added clarification that we used the spatial and temporal patterns of species’

presence determined in previous studies. “Instead of relying on these automated detection

methods, we used the spatial and temporal patterns determined in previous studies

(20, 29) to determine which species were present and avoided sites and seasons in which

there was likely to be overlap in presence.” (line 154-157)

L117- By "In recordings from 21 hours on 16 days" you mean "In a total of 21 hours of recordings from 16 days.."
Corrected.

L122 - It should read "attenuata" instead of "attenuatte"
Corrected.

L133- Should read as "was analyzed" instead of "were analized.
Changed as suggested. Thank you.

L138 - in the version that I have, Figure 2 is repeated
Apologies. There is only one Figure 2 included.

L149 - This section needs to be further explained and developed. I understand that you compared the SPL levels of those whistles that reocurred at least once. So your N = 100? that's what you used for the GEE?. This needs further explaining in this section.
We have clarified our methods here and added “The characteristics of 100 randomly

selected signature whistles that re-occurred were modeled with the GEEs against the

sound levels in which they occurred.” (line 196-197)

154-155 - it should read as " Ambient sound levels were calculated in MATLAB (MathWorks,
Natick, Massachusetts, USA) as the relative broadband (up to 24 kHz, given the sampling rate of
48 kHz) root mean square (RMS) sound pressure level (SPL in dB re 1µPa ) during the recording in which the signature whistle occurred (two or five minutes in duration)"

This change has been made as suggested (line 190).

L306 - maybe a reference here to justify that 120dB is high. Southall et al has good research on this topic.

We have added a reference to the National Research Council’s 2005 paper on

biologically significant effects of noise.
